# PH motifs in PAR_1&2_ endow breast cancer growth

**DOI:** 10.1038/ncomms9853

**Published:** 2015-11-24

**Authors:** A. Kancharla, M. Maoz, M. Jaber, D. Agranovich, T. Peretz, S. Grisaru-Granovsky, B. Uziely, R. Bar-Shavit

**Affiliations:** 1 Sharett Institute of Oncology, Hadassah-Hebrew University Medical Center Jerusalem, Jerusalem 91120, Israel; 2 Department of Obstetrics and Gynecology, Hebrew-University, Shaare Zedek Jerusalem 91031, Israel

## Abstract

Although emerging roles of protease-activated receptor_1&2_ (PAR_1&2_) in cancer are recognized, their underlying signalling events are poorly understood. Here we show signal-binding motifs in PAR_1&2_ that are critical for breast cancer growth. This occurs via the association of the pleckstrin homology (PH) domain with Akt/PKB as a key signalling event of PARs. Other PH-domain signal-proteins such as Etk/Bmx and Vav_3_ also associate with PAR_1_ and PAR_2_ through their PH domains. PAR_1_ and PAR_2_ bind with priority to Etk/Bmx. A point mutation in PAR_2_, H349A, but not in R352A, abrogates PH-protein association and is sufficient to markedly reduce PAR_2_-instigated breast tumour growth *in vivo* and placental extravillous trophoblast (EVT) invasion *in vitro*. Similarly, the PAR_1_ mutant *hPar1-7A,* which is unable to bind the PH domain, reduces mammary tumours and EVT invasion, endowing these motifs with physiological significance and underscoring the importance of these previously unknown PAR_1_ and PAR_2_ PH-domain-binding motifs in both pathological and physiological invasion processes.

## Abstract (web summary)

Protease-activated-receptor 1 and 2 (PAR1 and PAR2) are key players in tumor growth. In this study, the authors identify PAR1 and PAR2 domains that bind oncogenic signalling proteins driving breast cancer progression *in vivo* and placental extravillous trophoblast invasion *in vitro*.

## Graphical abstract

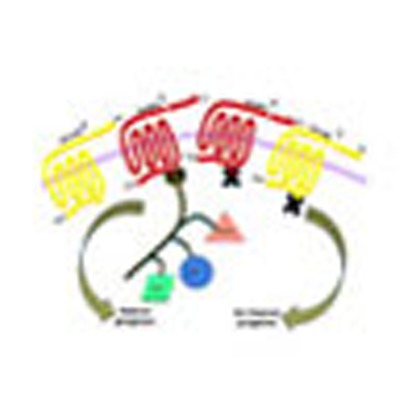

G protein-coupled receptors (GPCRs) are the largest family in mammalian cells, mediating a plethora of physiological responses^1^,^2^. Despite the fact that GPCRs emerge as oncogenes that regulate cancer-associated signalling networks, their role in tumour biology is not well understood. Indeed, large-scale genome analyses of multiple human tumours have uncovered novel GPCR alterations, as well as aberrant overexpression of GPCRs in cancer^3^,^4^. It is imperative to determine which of the GPCRs are cancer instigators rather than bystanders to enable identification of candidate genes for future targeted personalized medicine.

During cancer progression, normal epithelial organization is disrupted and cells are maintained outside their normal niches^5^,^6^. Both soluble and matrix-immobilized proteases are present in the dynamic and flexible microenvironment of a tumour and contribute to the process of cancer advancement^7^. One example is the activation of cell surface protease-activated receptors (PARs). Mammalian PARs are a subgroup of GPCRs that form a four-member family^8^,^9^,^10^,^11^,^12^. PAR_1_ and PAR_2_ play a central role in epithelial tumour growth in a variety of malignancies^13^,^14^,^15^,^16^. Whereas PAR_2_ is not considered a thrombin receptor (unlike PAR_1,3_ and_4_), the PAR_1_-tethered ligand SFLLRN is capable of transactivating PAR_2_ (refs 17, 18). Increasing evidence supports the notion that PAR_1_ and PAR_2_ exist in close proximity and act as one functional unit forming heterodimers^17^,^18^,^19^. Consistently, we have found that PAR_2_ plays a dominant role in PAR_1_–PAR_2_-instigated tumour activity^20^.

Among the protein modules that drive intermolecular interactions in cellular signalling, the pleckstrin homology (PH) domain is most common. PH domains are mainly recognized by their structural characteristics, and with a seven-stranded β-sandwich and a C-terminal α-helix^21^. While PH domains lack primary sequence similarity, their superfold assembly represents a particularly stable structural scaffold employed in many different functions^22^.

Here we describe PH-domain-binding motifs in PAR_1_ and PAR_2_ C-tails that are necessary for PAR-driven tumour growth and *time-limited* placental trophoblast invasion. We propose that these PH-domain-binding motifs may serve as an important molecular mechanism within the PAR signalling network and provide a platform for future drug therapy design.

## Results

### PAR_2_ associates with Akt/PKB via its PH domain

**Figure 1:**
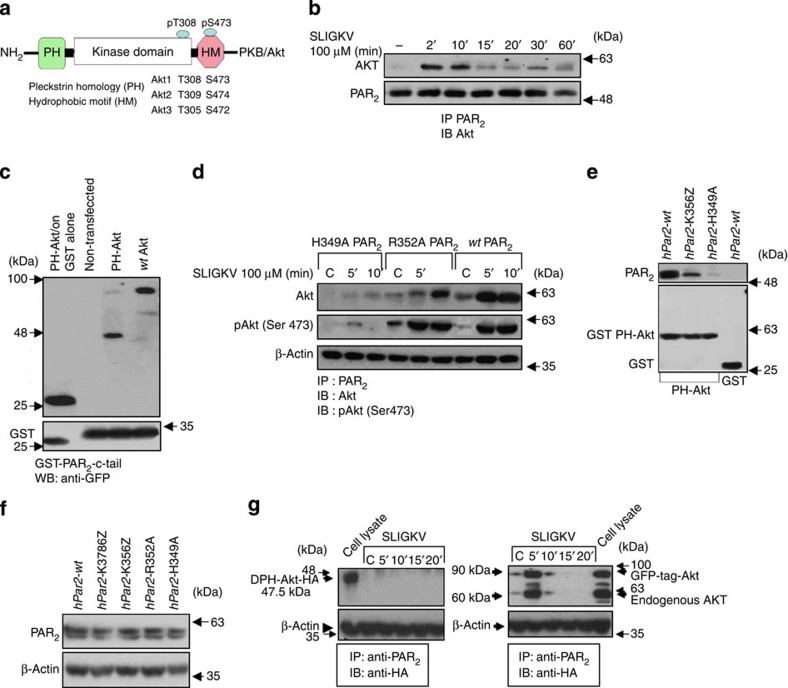
Akt/PKB associates with PAR_2_ C-tail via its PH domain. (**a**) Schematic presentation of Akt/PKB. (**b**) Immunoprecipitation (IP) analysis of PAR_2_ and Akt/PKB. HEK 293T cells were transfected with *wt hPar2*. IP was performed following PAR_2_ activation using anti PAR_2_ antibodies and immunoblotting with anti-Akt antibodies. Co-immunoprecipitation (Co-IP) was performed following SLIGKV PAR_2_ activation of *wt hPar2* at 2–10 min. (**c**) GST-PAR_2_-C-tail binds *wt* Akt or Akt-PH-domain module alone. HU nearly normal cells (naive cells not expresing endogenous PAR_2_) were transiently transfected or not with either GFP-*wt* Akt or GFP-PH-domain alone. Cell lysates were applied to the GST-PAR_2_ C-tail. Specific binding was seen following separation on SDS–PAGE and detection using anti-GFP antibodies. (**d**) PAR_2_ mutant H349A fails to associate with Akt. HU cells were transiently transfected either with *wt hPar2*, PAR_2_ mutant R352A or PAR_2_ mutant H349A. Cell lysates were immunoprecipitated following SLIGKV PAR_2_ activation using anti-PAR_2_ antibodies. Detection by western blot analyses of Akt PAR_2_ association was performed using anti-Akt antibodies. Phosphorylation of Akt was detected using anti-phospho Ser473 antibodies. Where indicated, IP detection of PAR_2_ was carried out using anti PAR_2_ SAM11 ab (1 μg per assay) at 1:500 dilution. (**e**) PAR_2_ mutant H349A fails to associate with GST-Akt-PH domain. Application of HU cells following transient transfection with the indicated constructs (for example, *wt hPar2*, a short *hPar2* C-tail-K356Z and the *hPar2* mutant H349A) on columns of GST-Akt-PH domain, resulted in effective association as detected by western blot analysis with the *wt* and PAR_2_-K356Z constructs. No binding was seen in the presence of the PAR_2_ point mutation H349A. (**f**) Expression of PAR_2_ constructs. HU cells were transiently transfected with the indicated PAR_2_ constructs. Level of construct expression is detected by Western blot analysis, using anti PAR_2_ antibodies. (**g**) Delta PH-Akt does not associate with PAR_2_ C-tail. HU cells were transiently transfected with *YFP*-*hPar2* construct and either GFP-*wt*-Akt or myr-Akt delta 4–129 (PH domain) HA-tag. IP was performed following PAR_2_ SLIGKV activation, using anti-PAR_2_ antibodies and immunoblotting with anti-Akt antibodies. While co-association was observed in cells transfected with GFP-*wt* Akt, in-contrast, no association was seen following SLIGKV PAR_2_ activation in cells transfected with the myr-Akt delta 4–129 (PH-domain) HA-tag plasmid. Control for protein loading is shown by levels of β-actin.

To identify a key signalling partner that plays a role in PAR_2_-driven tumour growth (Supplementary Fig. 1), we analysed the interaction between PAR_2_ and Akt/PKB, a serine/threonine protein kinase that plays a pivotal role in tumour cell survival, proliferation and invasiveness^23^,^24^. HEK-293T cells were transiently transfected with *hPar2* and cell lysates, before and after SLIGKV-PAR_2_ activation, were immunoprecipitated with anti-PAR_2_ antibodies and analysed for Akt/PKB co-association. A tight association was observed after 2–10 min of activation, which declined thereafter (Fig. 1a,b). This interaction takes place via the binding of PAR_2_ C-tail with the PH domain of Akt/PKB, as evaluated by the GST-PAR_2_ C-tail pull-down assay (Fig. 1c). Akt/PKB also co-associates with PAR_1_ via its PH domain (Supplementary Fig. 2).

To conclusively identify the critical amino acids involved in this association, point mutations were inserted in the PAR_2_ C-tail downstream of the membrane-anchoring site (R352A and H349A, Supplementary Table 1). While binding was observed with mutant R352A similar to *wt* PAR_2_, no association was seen with H349A (Fig. 1d). The PAR_2_-bound Akt was functionally active, as demonstrated by high phosphorylation levels of Ser473 (Fig. 1d). Importantly, introduction of these mutants did not alter the cell distribution of PAR_2_, as shown by the analysis of the cell surface expression, although truncated *hPar2* was expressed to somewhat a lower level (Supplementary Fig. 3). When cell lysates overexpressing either *wt hPar2* or a short deleted PAR_2_ C-tail construct (*hPar2-*K356Z) and mutant *hPar2*-H349A were applied to GST-PH-Akt columns, a potent binding association with *wt hPar2* and with *hPar2* K356Z was obtained. No binding was observed when cell lysates expressing mutant PAR_2_ H349A were applied, or when *wt hPar2* was applied to GST beads alone (Fig. 1e). Levels of the various constructs expression are shown in Fig. 1f. When an Akt construct that was devoid of the PH domain was utilized, no association between PAR_2_ and Akt took place (Fig. 1g). This provides support for the role of the PH domain of Akt in the binding association between PAR_2_ and Akt. Furthermore, the mutant *hPar2*-H349A effectively inhibited cell migration and wound closure in a similar way to truncated *hPar2*. In contrast, activation of *wt hPar2* potently induced migration and closure of the wound by 72 h. SLIGKV activation of mutant *hPar2*-R352A induced migration, although at a slower rate compared with *wt hPar2* (Supplementary Fig. 4).

### Other PH-domain signal proteins associate with PAR_1/2_

**Figure 2:**
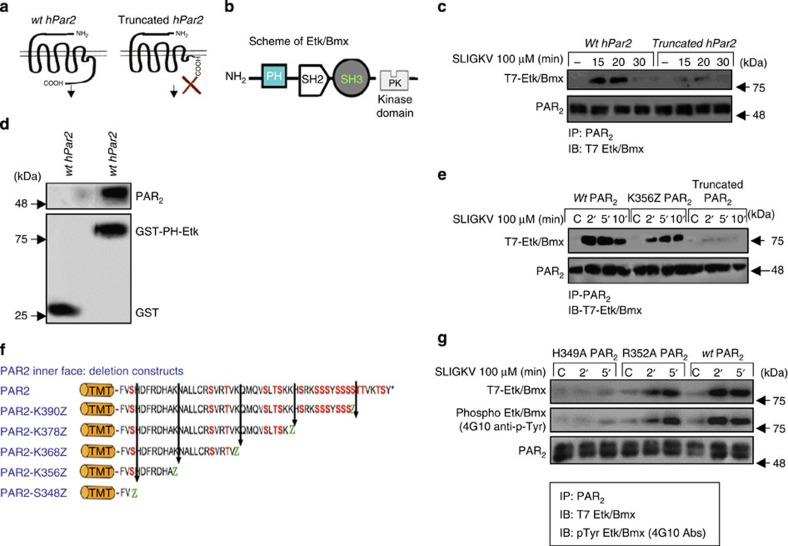
Association between PAR_2_ and Etk/Bmx via its PH domain. (**a**) Schematic presentation of *wt* and truncated PAR_2_. (**b**) Scheme of Etk/Bmx. (**c**) HEK293T cells were transfected with T7 Etk/Bmx and either *wt hPar2* or a truncated form of *hPar2* plasmids. IP was performed following PAR_2_ activation using anti-PAR_2_ antibodies and immunoblotting with anti-T7 for Etk/Bmx detection (T7 tagged Etk/Bmx) of a Western blot. Effective co-immunoprecipitation was seen following SLIGKV PAR_2_ activation of *wt hPar2* but not with a truncated form of *hPar2*. (**d**) PAR_2_ binding to GST-PH-Etk/bmx domain. Cell lysates expressing PAR_2_ were applied on either GST-PH-Etk/Bmx or GST beads alone, specific binding was seen on the GST-PH-Etk/Bmx beads but not on the GST beads, as detected by Western blot analyses. (**e**) Co-IP between *wt* PAR_2_ and PAR_2_ deleted constructs. HEK293T cells were transiently transfected with the various PAR_2_ constructs. While effective association was seen following SLIGKV PAR_2_ activation in the *wt* PAR_2_ and the shortest C-tail PAR_2_ K356Z deleted construct, no association was observed in the presence of the truncated form of PAR_2_, which was devoid of the entire C-tail. (**f**) Schematic representation of PAR_2_ C-tail deleted constructs. (**g**) PAR_2_ mutant H349A fails to bind Etk/Bmx. HU cells were transiently transfected with either *wt hPar2*, PAR_2_ mutant R352A, or PAR_2_ mutant H349A. Cell lysates were immunoprecipitated following SLIGKV PAR_2_ activation with anti-PAR_2_ antibodies. Detection for Etk/Bmx PAR_2_ association was carried out by anti-T7 antibodies. Tyr-phosphorylation of bound Etk/Bmx was detected using anti-4G10 antibodies for the detection of phosphotyrosine residues. Levels of PAR_2_ are shown as control. When indicated, detection of PAR_2_ was carried out using either SAM 11ab (Santa Cruz Biotechnology) at1:500 dilution.

We next examined the possibility that additional signal proteins carrying a PH domain are capable of association with PAR. ExPASy proteomics was used to identify a wide panel of PH-domain-containing proteins. Among others, the signal proteins Etk/Bmx, Akt, Vav, SOS1 and GAB1 were found to carry this domain. We chose to focus on two signalling proteins, Etk/Bmx and Vav_3_. The interaction between PAR_2_ and Etk/Bmx, a member of the nonreceptor tyrosine kinase family encoded by the *BMX* gene^25^,^26^ was examined. We previously examined the interaction between Etk/Bmx and PAR_1_ (ref. 27). Distinct association between PAR_2_ and Etk/Bmx was observed at 15–20 min, which declined thereafter (Fig. 2a–c). In contrast, no binding was obtained when a truncated form of *hPar2*, devoid of the entire cytoplasmic tail, was ectopically expressed in the cells. This association takes place via the binding of the PAR_2_ C-tail with the PH domain of Etk/Bmx, as evaluated by the GST-PH-Etk/Bmx pull-down assay (Fig. 2d).

We then determined the minimal PH-domain-binding region within the PAR_2_ C-tail sequence. For this purpose, we prepared deleted PAR_2_ C-tail constructs. Effective co-association with the Etk/Bmx PH domain was observed with the shortest C-tail construct *hPar2*-K356Z (Fig. 2e,f), and similar with the *wt hPar2* construct. Following insertion of mutations into the short K356Z region, we found that in HEK 293T cells overexpressing either R352A or H349A mutants no association was seen with the mutant H349A; however, the R352A mutant did associate with the Etk/Bmx PH domain (Fig. 2g and supplementary Fig. 5A). PAR_2_ C-tail-bound Etk/Bmx was functionally active, allowing downstream signal association, as demonstrated by induced Tyr-phosphorylation levels (Fig. 2g). We therefore conclude that the amino acid histidine at position 349 is critical for the association of PAR_2_ with the Etk/Bmx PH domain, as was seen above with Akt/PKB.

**Figure 3:**
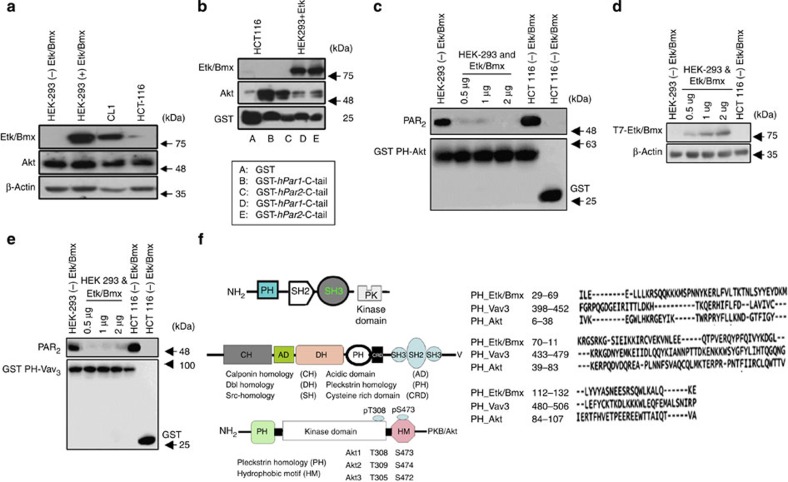
PAR_1&2_ C-tails associate with Etk/Bmx or Akt-PH domains. (**a**) Western blot analysis of Akt and Etk/Bmx in cell lines. Cell lysates of the indicated cell lines were analysed by western blot analysis. Application of anti-Etk/Bmx or anti-Akt antibodies showed that endogenous Etk/Bmx was present only in CL1 cells, an aggressive prostate cancer cell line, or following ectopic overexpression in HEK 293T cells. In contrast, Akt was abundantly present in all the cell lines analysed. (**b**) PAR_1_- and PAR_2_-GST-C-tails associate with Etk/Bmx and Akt. Specific association of the Akt-PH domain was seen in cells that do not express Etk/Bmx (for example, HEK 293T and HCT116 cells). HEK293T cell lysates overexpressing *etk/bmx* plasmid concentrations (0.5–2 μg) showed no association with the Akt-PH domain. Application of HEK293T cells transfected to overexpress Etk/Bmx showed effective immobilization on both PAR_1_ and PAR_2_ C-tails. Application of HCT116 colon cancer cells that do not express Etk/Bmx showed a marked association with Akt. (**c**) PAR_2_ binds GST-PH-Akt. Specific association of the Akt-PH domain was seen in cells that do not express Etk/Bmx (for example, HEK 293T and HCT116 cells). HEK293T cell lysates overexpressing *etk/bmx* plasmid concentrations (0.5–2 μg) showed no association with the Akt-PH domain. (**d**) Western blot analysis. Levels of T7-Etk/Bmx following increased *etk/bmx* plasmid transfection are shown. β-actin was used as a loading control. (**e**) PAR_2_ binds GST-PH-Vav3. Specific association of the Vav_3_-PH domain was seen in cells without Etk/Bmx expression (for example, HEK 293T and HCT116 cells). HEK293T cell lysates overexpressing *etk/bmx* plasmid concentrations (0.5–2 μg) showed no association with the Vav_3_-PH domain. When indicated, detection of PAR_2_ was carried out using either SAM 11ab (Santa Cruz Biotechnology) at 1:500 dilution. (**f**) Alignment PH-domain sequences of Etk/Bmx, Vav_3_ and Akt. (**d**) PAR_2_ binds GST**-**PH-Vav3. Multiple sequence alignment of PH-Etk/Bmx, PH-Vav_3_ and PH-Akt was performed using T-coffee (available at http://www.ebi.ac.uk/Tools/msa/tcoffee).

We observed that, while Akt was abundantly expressed in the cancer cell lines examined, Etk/Bmx expression was restricted to CL1, a prostate cancer cell line (Fig. 3a). Lysates of cells expressing endogenous or transfected Etk/Bmx, as well as lysates of cells that do not express Etk/Bmx, were loaded on glutathione *S*-transferase beads fused to the individual PAR C-tails (either PAR_1_ or PAR_2_) for a pull-down assay. Specific association of Etk/Bmx with both GST-PAR_1_ and GST-PAR_2_ C-tails was observed (Fig. 3b). In contrast, in HCT-116 cells that lack endogenous Etk/Bmx, effective association with Akt was observed for both PAR_1_ and PAR_2_ C-tails (Fig. 3b).

### Hierarchy of binding

We also examined the priority of binding for Etk/Bmx by adding appropriately modified cell lysates to GST beads fused either to PH-Akt (Fig. 3c,d) or to PH-Vav_3_ columns (Fig. 3E). Vav_3_ oncogene, a guanine nucleotide exchange factor (GEF) of the Rho family GTPases, belongs to the Vav protein family^28^,^29^,^30^ and is ubiquitously expressed in breast and prostate cancers. Although all Vav family proteins have similar structural features, they display different tissue expression patterns. Vav_1_ is primarily expressed in haematopoietic lineages, while Vav_2_ is ubiquitously expressed. Vav_3_ has a broad but distinct expression profile compared with that of Vav_2_. We showed binding with Vav_3_ by a pull-down assay using PH-Vav_3_ columns (Fig. 3e and Supplementary Fig 5B). Before the GST-binding assay, the applied lysates of tumour cell lines were analysed for endogenous expression of either Akt or Etk/Bmx or both. These experiments showed that PAR_1_ and PAR_2_ have priority for binding with the Etk/Bmx PH domain over Akt or Vav_3_. Only when Etk/Bmx was absent did another PH-domain signal protein such as Akt bind with both PAR_1_ and PAR_2_ C-tails.

### PH-Akt/Vav_3_/Etk/Bmx bind to the same site on the PAR_2_ C-tail

HEK293T cells overexpressing PAR_2_ mutants (R352A and H349A) or PAR_2_ deletion constructs were added to GST-PH-Etk/Bmx, GST-PH-Akt or GST-PH-Vav_3_. While a specific association was observed with *wt hPar2*, *hPar2 K368Z* and *hPar2 K356Z*, as well as the mutant R352A, no binding was seen when H349A was added to GST-PH-Etk/Bmx, GST-PH-Akt or GST-PH-Vav_3_ (Fig. 3a–d). Histidine at position 349 is thus a critical amino acid for PH-signal proteins. Similarly, the same binding region in the PAR_1_ C-tail was found for the PH-domain signal proteins tested^27^. Sequence alignment of PH-Etk/Bmx, PH-Vav_3_ and PH-Akt showed a high number of different primary sequences (Fig. 3f).

### Characterization of PH-Akt/Etk/Bmx binding

**Figure 4:**
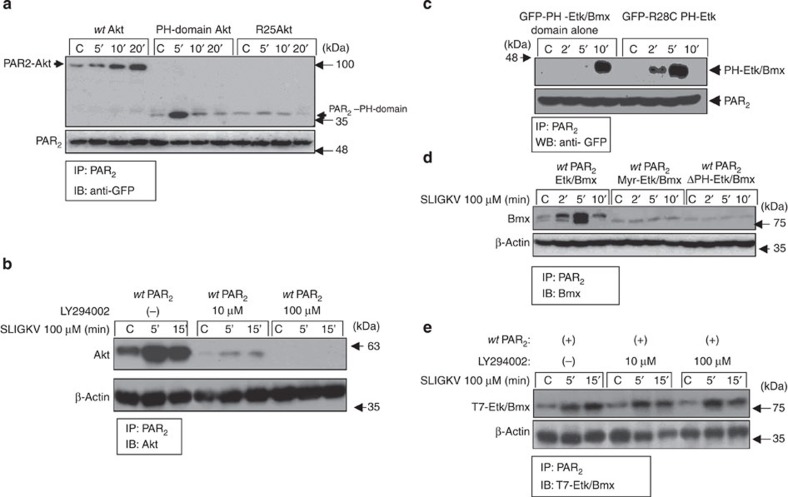
Mechanisms of Akt-PH and Etk/Bmx-PH domain binding to PAR_2_. (**a**) PH-Akt module binds PAR_2_-C-tail but not mutant R25C. HU cells were transiently transfected with *hPar2* construct and either with GFP-*wt*-Akt, GFP-PH-Akt domain alone or GFP-R25C. Immunoprecipitation of cell lysates following PAR_2_ activation was carried out using anti-PAR_2_ antibodies. Detection of either *wt* Akt or Akt-PH domain alone associated with PAR_2_ was performed with anti-GFP. While *wt* GFP-Akt and GFP-PH domain alone were shown to bind PAR_2_, no binding was obtained when the mutant R25C of low lipid-binding-affinity was present. (**b**) PI3K inhibitor (LY 294002) inhibits binding of Akt to PAR_2_. Treatment of HU cells with increasing concentrations of LY294002 following transient transfection with *hPar2* and SLIGKV activation was performed. Cell lysates were immunoprecipitated with anti-PAR_2_ antibodies, and anti-Akt was used to assess the association of Akt with the PAR_2_ C-tail. A potent inhibition of Akt-PAR_2_ association was observed in the presence of LY294002. (**c**) GFP-PH-Etk/Bmx module and mutant R28C bind PAR_2_. HU cells were transiently transfected with *hPar2* construct and with either GFP-PH-Etk/Bmx domain alone or GFP-R28C Etk/Bmx mutant with low lipid-binding affinity. Immunoprecipitation of cell lysates following PAR_2_ activation was performed using anti-PAR_2_ antibodies. Anti-GFP was used to detect the intact Etk/Bmx-PH domain alone associated with PAR_2_ and the mutant R28C. Both constructs bind PAR_2_ potently. (**d**) Co-IP between *wt* and Etk/Bmx modulation constructs. Co-IP analyses performed utilizing HU cells transiently transfected with either *wt hPar2*, *wt* Etk/Bmx, Myr-dPH construct or TH-SH2-SH3-KD. No association between Etk/Bmx and PAR_2_ was observed either with Myr-dPH constructs or TH-SH2-SH3-KD construct deleted for the PH domain. In comparison, effective association was obtained with *wt* Etk/Bmx. (**e**) LY294002 does not inhibit association of Etk/Bmx with PAR_2_. Treatment of HU cells with increasing concentrations of LY294002 following transient transfection with both T7-*etk/bmx* and *hPar2* and SLIGKV activation was performed. Cell lysates were immunoprecipitated with anti-PAR_2_ antibodies and Etk/Bmx- PAR_2_ association byanti-T7.

There have been a considerable number of reports about the proposed function of Akt PH domain lipid membrane recruitment and AKT activation^31^. AKT is a known phospholipid-binding serine/threonine kinase, and a key component of the phosphoinositide 3-kinase (PI3K) cell survival-signalling pathway that is aberrantly activated in many human cancers^32^. We observed distinct binding of either *wt* Akt or the GFP-Akt PH domain module alone with the PAR_2_ C-tail. In contrast, the Akt PH domain mutant R25C, which has low lipid-binding affinity, failed to associate with PAR_2_ (Fig. 4a). In addition, the application of LY294002, a PI3K inhibitor, completely abrogated the otherwise potent association between PAR_2_ and Akt (Fig. 4b). The involvement of phosphatidylinositol (3,4,5)-trisphosphate (PtdIns(3,4,5)P3; PIP3) in the PAR_2_–Akt association is supported by the potent inhibition of PAR_2_-Akt binding in the presence of Ins (1,3,4,5) P4 (IP4) (Supplementary Fig. 6). We conclude that the Akt–PAR_2_ association involves membrane anchoring via the PH domain. On the other hand, both the PH domain mutant R28C of the Etk/Bmx PH domain, which is incapable of lipid binding, and the GFP-Etk/Bmx PH domain module alone, exhibited potent binding with PAR_2_ C-tail (Fig. 4c).

To better characterize the nature of binding between PAR_2_ and the Etk/Bmx PH domain, analyses of additional Etk/Bmx constructs were carried out. These constructs were an Etk/Bmx construct whose PH domain was replaced by a myristoyl group attached to the N-terminal portion, Myr-dPH, and a construct depleted of the PH-domain containing the kinase domain, SH3, and SH2 domains, TH-SH2-SH3-KD (refs 33, 34). On SLIGKV activation of PAR_2_, no association between Myr-dPH Etk/Bmx and PAR_2_ was observed (Fig. 4d). Similarly, no interaction was seen when a deleted PH domain TH-SH2-SH3-KD construct was used (Fig. 4d). Accordingly, the presence of LY294002 PI3K inhibitor did not abrogate the association between PAR_2_ and Etk/Bmx (Fig. 4e). Hence, the Etk/Bmx PH domain association is completely lipid-independent and binds via a protein–protein interaction. This stands in contrast to Akt PH domain association with PAR_2_ C-tail, which is membrane lipid-dependent.

### PAR_2_ PH-domain-binding site is critical for EVT invasion

**Figure 5:**
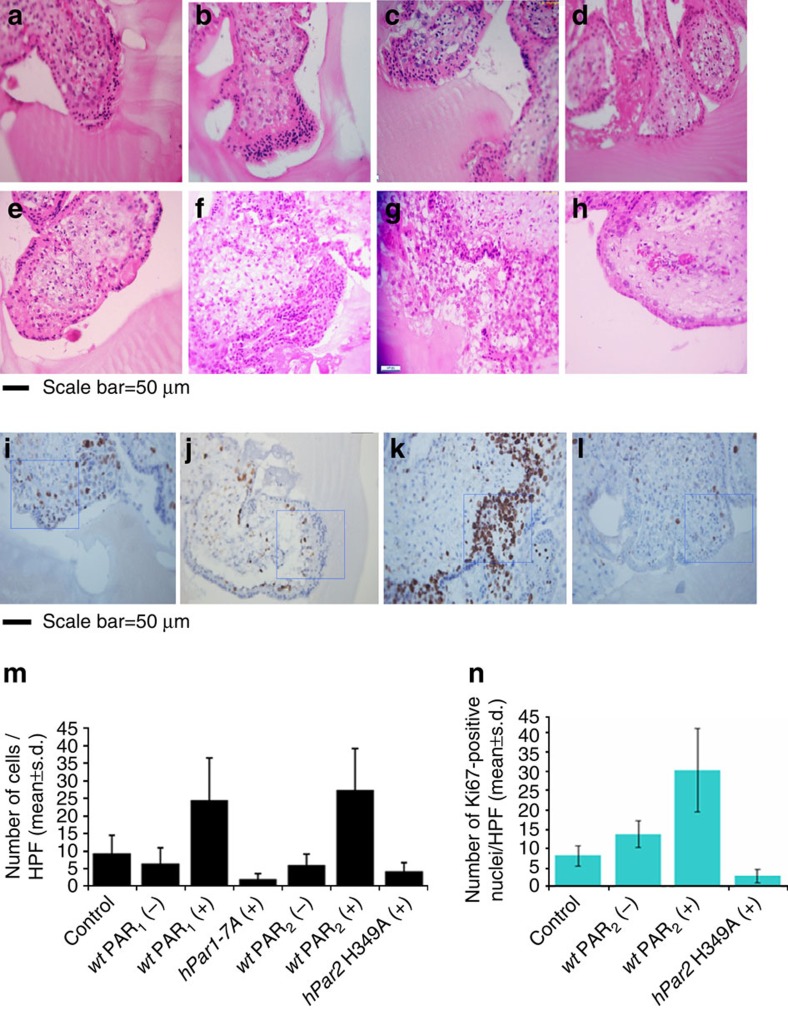
Placenta–EVT organ culture invasion. (**a**–**h**) Morphology of EVT column growth. Placental explants from the EVT–Matrigel cylinder cast after H&E staining (magnification × 40). Serial 5-μm sections of the Matrigel cylinder were embedded in paraffin blocks prepared for each of the treatment. Images of representative H&E-stained sections at 60 μm depth are presented. (**a**) Untreated (control), (**b**) *wt hPar1*-infected explants, (**c**) thrombin (1 U ml^−1^) activation, (**d**) *hPar1* 7A mutant and thrombin activation, (**e**) control, untreated, (**f**) *wt hPar2*, (**g**) *wt hPar2* and SLIKGV (100 μM) activation, (**h**) *hPar2* mutant H349A and SLIKGV (100 μM) activation. Note the increase in EVT-migrating cells and cell mass at the tip of the villi following either PAR_1_ or PAR_2_ activation. In contrast, a blunt end of the villi without sprouting cells was seen at the villi tip in the presence of mutants following activation treatments. The experiment was terminated 72 h after EVT treatment. Each experiment was performed using three different placentae, in triplicate. (**i**–**l**) Immunohistochemical staining of Ki67. Ki67 levels (measure of proliferation) were evaluated in (**i**) untreated (control), (**j**) wt hPar_2_, (**k**) wt hPar_2_ and SLIKGV (100mM) activation and (**l**) hPar_2_ mutant H349A and SLIKGV (100mM) activation. (**m**) Quantification of EVT outgrowth. Cell number was determined at a 60-μm depth of invasion as shown by the histogram mean values. Cells (not the villous compartment) were counted per high-power field (HPF) and expressed as mean±s.e.m. *Post hoc* evaluation of multiple comparisons (ANOVA Tukey HSD) showed a *P* value of 0.0001 within groups. The mean difference was significant at the 0.05 level. ANOVA evaluation was performed with the IBM/SPSS 20.0 software (Chicago, IL, USA). (**n**) Quantification of Ki67. Histogram of the mean values of Ki67 nuclear staining is shown. Cell nuclei were counted per HPF and expressed as mean±s.e.m. *Post hoc* evaluation of multiple comparisons (ANOVA Tukey HSD) showed a *P* value of 0.0003 within groups. The mean difference was significant at the 0.07 level. ANOVA evaluation was performed with the IBM/SPSS 20.0 software.

Establishment of human pregnancy involves the well-orchestrated invasion to the uterus wall of specialized cells termed extravillous trophoblasts (EVT). We have utilized an *in vitro* model based on isolation of villi from early gestation human placentae plated on Matrigel^20^,^35^,^36^,^37^. Placental specimens were harvested at the end of the first trimester (10–12 weeks) and those expressing little or no endogenous PAR_1_ and PAR_2_ were selected. These cultures were transfected with lentiviral *wt hPar1*, viral *hPar1-7*A mutant, *wt hPar2*, or *hPar2* mutant H349A, and were activated by their respective ligands. The impact of PAR_1_ or PAR_2_ activation on *wt* and mutant forms was evaluated by comparing the depth of invasion. EVT invasion into the Matrigel reached a maximal invasion depth of 100 μm, as evaluated by 5-μm serial sections of the Matrigel cast. Histological analyses of haematoxylin and eosin (H&E) staining are shown in Fig. 5a–h. Significant inhibition in EVT cell invasion was seen in the presence of the mutants that are incapable of association with PH-domain signal protein/s (activated *hPar1-*7A or *hPar2* H349A, Fig. 5a, lanes d and h, respectively), and before activation (Fig. 5a, b and e). In contrast, a high level of invasion was observed following either TFLLRN PAR_1_ or SLIGKV PAR_2_ activation, with invasion reaching up to 100 μm. Highly proliferative EVT cells at the tip of the villi recapitulate outgrowth and depth of invasion. Nuclear staining of ki67 demonstrates the extent of proliferating cells following *wt hPar2* and the mutant forms following activation (Fig. 5i–l). The number of cells per high-power field for each treatment was evaluated at an equal level of invasion (60 μm), as shown in the representative histogram (Fig. 5m–n).

### PAR_2_ Histidine349 is critical for tumour growth *in vivo*

**Figure 6:**
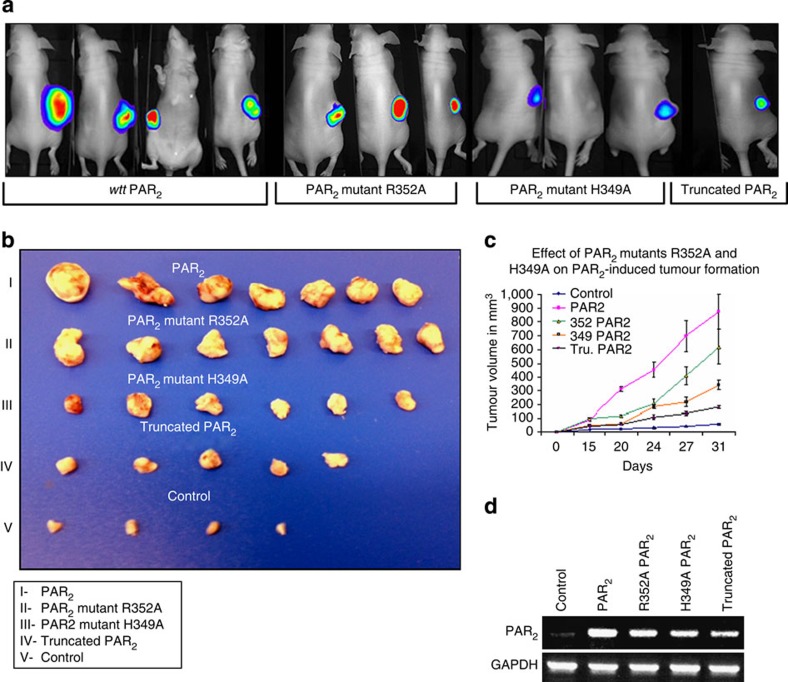
PAR_2_ PH-domain-binding sequence is essential for tumour growth *in vivo.* Stable clones of HU cells were prepared expressing luciferase and either *wt hPar2,* or a truncated *hPar2* (devoid of the cytoplasmic tail), or PAR_2_ mutant R352A, or PAR_2_ mutant H349A. The various clones (3 × 10^6^ cells) were inoculated subcutaneously in nude mice. (**a**) Representative bioluminescence images. Levels of tumour luciferase following various cell-clone inoculation treatments. (**b**) Tumour morphological appearance. At the end of the experiment (for example, within 31 days; I–V), mice were terminated and the tumours (I–V) were excised, measured and weighed. (**c**) Measurements of tumour volume. Tumours were weighed and measured for size at the indicated time points and tumour volume (mm^3^) was calculated. Error bars show s.d.; * *P*<0.005. Data shown are representative of three independent experiments. (**d**) Levels *wt* and modified PAR_2_ in the stable clones. Stable clones expressing the various *hPar2* constructs, either *wt hPar2* or truncated *hPar2, hPar2* mutant R352A or mutant H349A, are shown using PCR. GAPDH levels were used as the control housekeeping gene.

The physiological significance of the PAR_2_ PH domain motif is also demonstrated using a xenograft model of tumour growth (Fig. 6a–c). We generated stable clones of a nearly normal fibrocystic cell line HU that lacked expression of endogenous PAR_2_ but expressed a luciferase construct and one of the following plasmids: *wt* PAR_2_, a truncated PAR_2_, and PAR_2_ mutants; R352A; or mutant R349A (Fig. 6d). The stable clones were inoculated subcutaneously into nude mice and were analysed for tumour growth. While large and vascularized tumours developed in mice inoculated with *wt* PAR_2_, no to minimal change in tumour size was seen in mutant R352A, and markedly smaller tumours were observed with the PAR_2_ mutant H349A, compared with PAR_2_-truncated clones (Fig. 6a–c). As mentioned above, the PAR_2_ R352A mutant did not abrogate PAR_2_ association with Etk/Bmx, Akt and the PH domain of Vav_3_ (Fig. 3c,d). In contrast, mutant H349A effectively abrogated the PAR_2_ association with all PH-domain signal proteins tested; hence, His (H) at position 349 is a critical amino acid for binding to all proposed downstream PH-domain signal proteins. Approximately 20-fold larger tumours were observed in mice inoculated with *wt hPar2* cells (825 mm^3^; *P*<0.0007) as compared with those in mice inoculated with control cells (40 mm^3^). In a Matrigel invasion assay, *wt* PAR_2_ consistently elicited a significantly higher level of HU cell invasion following PAR_2_ activation. In contrast, in the presence of PAR_2_ H349A, no Matrigel invasion was observed, comparable to levels in control mice, regardless of SLIGKV activation (Supplementary Fig. 7). Likewise, when a mutated form of *hPar1* (*hPar1-7A*) incapable of associating with the PH domain of Etk/Bmx (*hPar1-7A*) was analysed in a murine xenograft mammary model *in vivo*, a dramatic reduction in the otherwise large tumours was observed (Supplementary Figs 8 and 9).

It is interesting to note that neutrophil elastase (NE) is incapable of inducing the PAR_2_-PH-Etk/Bmx association (Supplementary Fig. 10), while association of PAR_1_ to either PH-Etk/Bmx or PH-Akt is elicited by NE (Supplementary Fig. 11).

## Discussion

PH domains are conserved protein motifs present in diverse signal-transducing proteins. They are known to be versatile modules in protein–protein interaction platforms in a plethora of physiological events^38^,^39^. We describe binding motifs capable of selective association with PH domains of Etk/Bmx, AKT and Vav_3_ in the C-tails of mammalian PAR_1_ and PAR_2_. These binding motifs are necessary for tumour development as well as physiological placental EVT–uterus interactions. Mutations inserted to the PAR_1_ PH domain (Supplementary Figs 7 and 8) and point mutation at H349A in PAR_2_ markedly attenuated xenograft tumour growth in a murine model of cell migration (Supplementary Fig. 4) and of placental–trophoblast time-limited invasion.

Despite the fact that sequence identity between PH domains is limited, their tertiary structures are strikingly similar. Although little is known about the molecular basis of PH-domain function, several lines of evidence indicate that these domains are critical for receptor activity. For example, coupling of insulin receptor and insulin receptor substrate, IRS-1, depends in part on the IRS-1 PH domain^40^,^41^. The PH domain of β-adrenergic receptor kinase is required for its interaction with the βγ-subunits of heterotrimeric G-proteins^42^.

Whereas many studies have addressed PH-domain-phospholipid-binding properties, protein–protein interactions are only now being investigated^43^. The PH domain of oncogenic Dbl mediates targeting to the cytoskeletal matrix and was found to be necessary for oncogenic transformation^44^. The PH domains in guanine nucleotide-binding proteins are essential for C-terminal association with the Dbl homology (DH) RhoGEF catalyst. Furthermore, phosphoinositol-lipid-binding PH sites are effective modules for both small guanine nucleotide-binding proteins^45^,^46^ and Gα-subunits^47^. Thus, both lipid-binding capabilities and protein–protein interactions play roles in PH-domain module interactions.

AKT activation is driven by binding of the PH domain to PIP3 or PIP2 for membrane localization, followed by the phosphorylation of serine 473 and threonine 308 (ref. 48). In addition, the PH domain plays a critical regulatory role in AKT function, and mutations disrupting the PH-domain function are apparently important in physiological and disease processes. For example, in a drosophila model, PIP3 levels are lethal when PTEN is lacking. Rescue survival is possible only if the drosophila AKT PH domain is inactive and incapable of binding lipid^49^. This indicates that AKT is a critical target, activated by increased PIP3, for a second lipid messenger pathway. A point mutation introduced into the PH-domain lipid-binding pocket of AKT1, whereby arginine is replaced by cysteine at amino acid 25 (AKT1R25C), results in low-affinity AKT-phospholipid binding, with both inhibited recruitment of AKT to the membrane and association with PAR_2_ (ref. 50). In another lipid-binding pocket mutation of the AKT–PH domain, E17K alters interactions of the pocket and activates AKT1 by means of pathological localization to the plasma membrane^51^. This mechanism suggests a direct role for AKT1 in human cancer and adds to the known genetic alterations that promote oncogenesis through the phosphatidylinositol-3-OH kinase/AKT pathway. Our data demonstrate that PAR_1_ and PAR_2_ harness the AKT pathway via binding to their PH domains, thus enabling their downstream network signalling.

It is well established that PIP3 functions to activate AKT via binding to the PH-domain-mediating translocation to the plasma membrane for the appropriate downstream signalling. Ins (1,4,5)P3 (IP3) however can be converted to IP4 by a family of inositol trisphosphate kinases. Once generated, IP4 can act as a soluble analogue of PIP3 and thereby negatively regulates PIP3 AKT-PH signalling. Indeed, our data show that potent inhibition of the association between PAR_2_ and PH-Akt that requires PIP3 is observed in the presence of increased IP4 concentrations (Supplementary Fig. 6). This regulation is similar to that of other diverse critical decision processes downstream of many receptors. Specific examples are the negative regulation of neutrophil signalling and chemotaxis^52^, neutrophil survival and bacteria killing by neutrophils, of overall importance for neutrophil function and innate immunity^53^.

Naturally occurring mutations in the PH domain of Bruton's tyrosine kinase interfere with phosphoinositide-binding properties and with X-linked agammaglobulinaemia in humans and X-linked immunodeficiency in mice^54^. Here we show that, in contrast to Akt PH domain lipid-binding dependency, the PH domain of Etk/Bmx binds PAR_2_ in a phospholipid-independent manner. This is based on the strong PAR_2_-Etk/Bmx association with the mutant that is incapable of binding lipids, and on the lack of association of modified Etk/Bmx constructs, in which the PH domain was replaced with a myristoyl group or with a depleted PH domain construct. This finding is in line with other Etk/Bmx protein–protein interactions, such as binding to the FAK-FERM domain^55^. It was similarly demonstrated that the PH domain of an Etk/Bmx lipid-binding-deficient mutant retained potent FAK-binding capability, indicating that the regulation and association of Etk/Bmx by FAK is independent of lipid-binding activity of the PH domain.

Vav_3_ proto-oncogene is a member of Vav belonging to the Dbl family of GEF for the Rho family of GTP-binding proteins. The characteristic structural domains of the family include an N-terminal calponin homology domain, an acidic region, a DH domain and a PH domain, among others. Generally, in these protein families the PH domain is located at the C-terminal DH helix and contributes to the support of GEF activity^56^,^57^. It is possible, but remains to be shown, that the preference for Etk/Bmx association with PARs over the Akt or Vav_3_ PH domain is due to direct protein–protein interactions, independent of lipid involvement, although the nature of the PH-Vav_3_ interaction remains to be fully determined.

Overall, regardless of unusually different primary sequences, PH domains share a conserved fold made up of a b-barrel composed of two roughly perpendicular, antiparallel beta-sheets and a C-terminal alpha amphipathic helix. On binding to phosphatidylinositol lipids, a key membrane constituent, PH domains act in the recruiting of proteins to the cell membrane. A large number of PH domains, however, have poor affinity for phosphoinositides and, in fact, function as protein-binding domains. Hence, PH domains are involved in the formation of signalling complexes involved in cell fate decisions and require precise temporal and spatial control. They may employ multiple biologically optimized interaction surfaces that together form a cooperative signalling device.

As for the hierarchy, the best interaction between PAR_1&2_ C-tails and PH domains takes place via protein–protein interaction, as with Etk/Bmx. However, since these interactions are of high importance, a back-up system is also available in cases when Etk/Bmx is absent in a specific physiological context, for example, in a PH-Akt association.

In summary, PH motifs for binding associations, either with lipids that are located within cellular membranes, or via protein–protein interactions, exemplify how the interplay between distinct motifs in a signal protein not only support transmission of a biochemical signal but also ensure a robust response to developmental cues, at precisely the right time, and with sufficient specificity to safeguard against premature and hence disastrous induction of cell fate change.

Biased signalling at GPCRs has redefined classical concepts in receptor pharmacology, not only highlighting the depth of signalling diversity within the GPCR system but also offering possibilities for more effective therapeutics^58^,^59^. We have now explored NE's role in determining the consequences of PH-Etk/Bmx-binding association with either PAR_1_ or PAR_2_. NE did not elicit the association of either of the PH-domain signal proteins analysed, Etk/BMX and Akt, with PAR_2_ (Supplementary Fig. 10). In contrast, NE was capable of eliciting PAR_1_ association with both PH-Etk/BMX and PH-Akt (Supplementary Fig. 11). This dichotomy can be explained by the NE cleavage site locations on PAR_2_ versus PAR_1_. While the main digestion site of the PAR_2_ N-terminal extracellular domain is rather far downstream from the canonical trypsin cleavage site and is allocated to residues F^(67)^ and ^(68)^SAS, NE cleaves PAR_1_ only four residues after the classical thrombin-cleaving site ^(41)^R/^(42)^SFLLRN, after L residue L^(45)^/R^(46)^NPNDKY. Our NE data are still preliminary and were not the focus of the current study. However, it appears that, at least with regard to PAR_1_, rather than disarming its function, the induced PH-Etk/BMx and/or PH-Akt association joins the MAPK activation previously demonstrated for NE but not for other neutrophil proteinases (for example, proteinase-3 and cathepsin G)^60^,^61^,^62^. The possibility that endogenous NE may be capable of instigating a key PAR_1_ signalling event, albeit to a diminished extent compared with classical activation, may be important. This proteinase is abundant in the surroundings of a tumour, providing support to the axis in terms of inflammation-induced tumour development.

Other PAR_2_ C-tail sequences have been described as having an impact on PAR_2_-induced cellular functions. A sequence region immediately downstream to the PH-domain-binding motif, allocated to residues 356–363, was found to affect PAR_2_-induced InsP_3_ accumulation, Ca^++^ mobilization and PYK activation. However, the role of this sequence is not clear. It was suggested that perhaps it has to do with the deletion of a palmitoylation site that is usually recognized by a cystein flanked by basic amino-acid residues, as is the case with residues 356–363 (ref. 63). In addition, Vogel's group has demonstrated crosstalk and physical interaction between PAR_2_ and TLR4, a member of the Toll-like receptor (TLR) family^64^. TLRs serve as important guards of the innate immune response through their ability to sense conserved pathogen-associated molecular patterns. The C-tail region sequence of PAR_2_, where the physical interaction with TLR4 takes place, is currently unknown and remains to be fully elucidated. Whether PAR_2_ PH-domain is critically involved in these interactions is an open question.

The three intracellular loops designated C1, C2 and C3 along with the C-tail, termed C4, are critical for class A GPCR signalling, and are recapitulated by interaction with the heterotrimeric G-proteins (for example, α, β and γ subunits)^65^. In general, C1 is the shortest in length, with relatively conserved length between family members, whereas the highest degree of variability is found in C3 and C4 (ref. 66). Ligand binding to GPCRs mediates a large conformational change most notably among others, in C2 and C3, which in turn promote activation of G-proteins by exchange of GDP for GTP on the Gα subunit^67^. An array of components composed of a lipid moiety (for example, palmitate, myristate and lithocholic acid) attached to a peptide that corresponds to an amino-acid segment of the cytoplasmic loops (C1, C2 or C3) or the C-terminal tail have shown however to affect all three intracellular loops and also C4, suggesting that all intracellular domains may be important for signal transduction. Within the C-terminal domain is the highly conserved eighth helix (H8) previously identified in rhodopsin^65^ and other Class A receptors, including PAR_1_ (ref. 68) and PAR_2_ (ref. 69). H8 is anchored to the membrane by palmitoylation of C-terminal cysteine residues. In fact, the PAR_1_ PH domain is localized within the H8 loop and was found to be confined to this region. Similarly, palmitoylation of PAR_2_ is necessary for post-translational modification and is required for efficient cell surface expression and desensitization of PAR_2_. Our data demonstrate for the first time that PH-domain-binding motifs in the PAR_1_ and PAR_2_ C-tails are critical signal-initiating sites. These findings define a molecular path in PAR-induced signalling networks. These sites are potential targets for future drug design. It is possible that other cancer ‘driver' GPCRs harbour PH-domain-binding motifs within their C-tails, which would contribute a more general significance to these sites. This possibility needs to be fully explored.

## Methods

### Cell culture

HEK-293T, MCF-7, HCT-116 and CL-1 cells (obtained from the American Type Culture Collection) were grown in DMEM. HU breast epithelial cells were generated by the late Dr Aviva Horowitz (member and friend of the Sharett Institute of Oncology, Hadassah-Hebrew University Medical Center, Jerusalem, Israel). The cells were grown in RPMI, supplemented with 1 mM L-glutamine, 50 μg ml^−1^ streptomycin, 50 U ml^−1^ penicillin (GIBCO-BRL, Gaithersburg, MD, USA) and 10% fetal calf serum (Biological Industries, Beit Haemek, Israel). Cells were maintained in a humidified incubator with 8% CO_2_ at 37 °C.

### Plasmids and transfection

A cDNA encoding wild-type human *Par2* was kindly provided by Professor Morley D. Hollenberg (Faculty of Medicine, University of Calgary, Alberta, Canada). Etk/Bmx viral vector and GST-PH-Etk/Bmx constructs were kindly provided by Dr Yun Qiu (Departments of Pharmacology and Experimental Therapeutics, University of Maryland School of Medicine, Baltimore, MD, USA). The GST-PH-Akt construct was kindly provided by Dr Brian A. Hemmings (Friedrich Miescher Institute, Basel, Switzerland). The GST-PH-Vav_3_ construct was kindly provided by Dr Shan Lu (University of Cincinnati College of Medicine, Cincinnati, OH, USA).

Cells were grown to 70–80% confluency and transfected with 0.1–3.5 μg of plasmid DNA in TransIT LT1 transfection reagent (Mirus Bio LLC, Madison, WI, USA) according to the manufacturer's instructions. Cells were collected 48 h after transfection and protein lysates/RNA were purified.

MCF-7, HU or HEK 293T were grown to 70–80% confluency and transfected with 1–2 μg of either *wt* human *hPar1* or *hPar2* or truncated *hPar2* (devoid of the cytoplasmic tail) cDNA, or with several *hPar2*-deleted constructs, or with a control pcDNA3 vector (Invitrogen, Carlsbad, CA, USA) using TransIT LT1 transfection reagent (Mirus Bio LLC). Transfected cells were selected with G418 (800 μg ml^−1^) to obtain stable populations of cells expressing *hPar1* and *hPar2*, or *hPar1* and truncated *hPar2*.

### PAR_1_ and PAR_2_ activation

Thrombin receptor-activating peptides TFLLRNPNDK for activation of PAR_1_ and SLIGKV for PAR_2_ were from GenScript (Piscataway, NJ, USA). Thrombin was obtained from OMRIX Bio Pharmaceutical (Ramat Gan, Israel).

### Gateway cloning

The *wt hPar1* (or) *hPar1 7A, wt hPar2* and mutant *hPar2* H349A-coding sequence was cloned into a neoR containing the lentiviral vector using the Gateway cloning system according to the manufacturer's instructions.

### Generation and titre of virus

Viral production was performed by co-transfecting neo HA *hPar1* vector orneo HA *hPar1 7A* vector (2 μg) and packaging vectors pCMVΔR8.91 (1.8 μg) and pMD2.VSVG (0.2 μg) into HEK293T cells using 12 μl TransIT LT1 transfection reagent (Mirus Bio LLC) in 100-mm plates. The resulting supernatant was collected after 48 and 72 h. The virus was recovered after ultracentrifugation for 1 h at 42,000 r.p.m. in a Beckman SW28 rotor (Beckman-Coulter, Brea, CA, USA). The resulting pellet was resuspended in medium to reach 100 × the initial viral concentration. Various *hPar2* constructs, *wt hPar2*, and truncated and deleted *hPar2* C-tail constructs, as well as mutants of *hPar2* were cloned into lentiviral vectors.

### Preparation of stable clones

MCF7 cells were infected with HA-*hPar1* virus or *hPar1*-7A virus. This was followed by selection using Geneticin G418 resistance (500 μg ml^−1^) to produce MCF7-*hPar1* and MCF7-*hPar1-7A* stable clones. To obtain MCF7-*hPar1-*Etk/Bmx and MCF7-*hPar1-7A*-Etk/Bmx stable clones, infection of Etk/Bmx virus was performed to the above-mentioned clones. Clone efficiency was evaluated using RT–PCR and western blotting. HU stable clones stably expressing *hPar2wt* and various deletion constructs, as well as mutants, were similarly generated.

### Mammary gland mouse model

Female athymic nude mice aged 6–7 weeks were pre-implanted subcutaneously with pellets containing 1.7 mg β-estradiol (60-day release, Innovative Research of America, Sarasota, FL, USA). Mouse mammary fat pads were then injected with 5 × 10^6^ MCF-7 cells stably expressing *hPar1 wt*, *hPar1 wt* and *Etk/Bmx*, *hPar1-7A* and *hPar1-7A* and *Etk/Bmx* constructs, or pcDNA3 control plasmid. Mice were monitored for tumour size by external calibre measurements (length and width) on days 10, 22, 25 and for up to 45 days if tumour burden allowed. Tumour volume (*V*) was calculated by *V*=*L* × *W*^2^ × 0.5, where *L* is length and *W* is width. At the end of the experiment, mice were killed and tumours were removed, weighed and fixed in formalin for histology. All animal experiments were approved by the animal committee of the Hebrew University (MD-09-11803-2).

### Histology

Tissue samples derived from mouse primary tumours were fixed with 4% formaldehyde in PBS, embedded in paraffin and sectioned (5 μm sections). After deparaffinization and rehydration, the sections were stained with H&E or were subjected to immunohistochemistry using specific antibodies.

### Immunohistochemistry

Paraffin-embedded slides were deparaffinized and incubated in 3% H_2_O_2_. Antigen unmasking was carried out by heating (20 min) in a microwave oven in 10 mM Tris buffer containing 1 mM EDTA. After blocking, slides were incubated with the following primary antibodies: PCNA (sc-56, Santa Cruz Biotechnology, Santa Cruz, CA, USA; dilution 1:200), β-catenin (C-2206, Sigma-Aldrich, St Louis, MO, USA; dilution 1:100) and caspase 3 (9661S, Cell Signaling Technology, Danvers, MA, USA; dilution 1:100) and diluted in CAS-Block (Invitrogen), or with CAS-Block alone, as a control. Appropriate secondary antibodies (Nichirei, Tokyo, Japan; dilution 1:1,000) were then added and the slides were incubated at room temperature for 30 min. Colour was developed using the 3,3′-diaminobenzidine (DAB) (Thermo Scientific, Walham, MA, USA) or the Zymed AEC substrate kit (Zymed Laboratories So., San Francisco, CA, USA), followed by counterstaining with Mayer's haematoxylin. Controls without addition of primary antibody showed low- or no background staining in all cases.

### Generation of PAR_2_ deletion constructs and mutants

PAR_2_ deletion mutants were generated with site-directed mutagenesis using QuikChange Lightning Site-Directed Mutagenesis Kit (Agilent Technologies Stratagene, Santa Clara, CA, USA) according to the manufacturer's instructions.

The primers used for insertion of stop codons, invariably along the *hPar2* C-tail, and for the generation of mutants R352A and H349A, are shown in Supplementary Table 2.

### Membrane solubilization

Cells were solubilized in lysis buffer containing 10 mM Tris-HCl (pH 8), 2.5 mM MgCl_2_, 5 mM KCl, 1 mM dithiothreitol, protease inhibitor mixture (1:100), 1 mM phenylmethylsulfonyl fluoride (PMSF) and 1 mM sodium orthovanadate (Sigma-Aldrich) for 30 min at 4 °C. After centrifugation at 12,000*g*, the supernatant containing the cytoplasmic fraction was collected and the pellet was resuspended with 1% Triton X-100, 150 mM NaCl and 50 mM Tris acetate (pH 8.2). The supernatants obtained after centrifugation at 12,000*g* contained the membrane fraction. This procedure facilitated enrichment of the membrane fraction.

### Western blot analysis and antibodies

Cells were solubilized for 30 min at 4 °C in lysis buffer containing 10 mM Tris-HCl, pH 7.4, 150 mM NaCl, 1 mM EDTA, 1% Triton X-100, a protease inhibitor cocktail (0.3 μM aprotinin, 1 mM PMSF; Sigma-Aldrich and 10 μM leupeptin). After centrifugation at 12,000*g* for 20 min at 4 °C, the supernatants were transferred and the protein content was measured. Lysates (50 μg) were loaded on a 10% SDS–PAGE gel followed by transfer to Immobilon-P membrane (EMD Millipore/Merck, Damstadt, Germany). Membranes were blocked and probed with the appropriate antibodies. Anti-HA for IP (1 μg per assay) was obtained from Santa Cruz Biotechnology, and for Western blot (HA.11mAb; 1:500 dilution) from Covance, Berkeley, USA. 1 μg/assay SAM11 antibody (Santa Cruz Biotechnology) was used for IP and a 1:500 dilution was used for Western blot detection of overexpressed *hPar2*. Rabbit anti GFP antibody, anti Akt antibody and anti phospho-Akt were obtained from Cell Signaling Technology and used at a dilution of 1:1,000. Mouse anti T7 antibody was obtained from Novagen Madison, WI; and used at a dilution of 1:10,000. Anti β-actin was purchased from Sigma-Aldrich and used at a dilution of 1: 5,000. Uncropped immunbloots and larger blot areas are represented in Supplementary Fig. 12

### Immunoprecipitation

Protein cell lysates (400 μg) were used for IP analysis. Mouse anti-PAR_2_ antibodies were added to the cell lysates. After overnight incubation, protein A-sepharose beads (Sigma-Aldrich) were added to the suspension, which was subsequently rotated at 4°C for 1 h. Elution of the reactive proteins was performed by resuspending the beads in protein sample buffer followed by boiling for 5 min. The supernatant was then resolved on a 10% SDS–polyacrylamide gel and was treated as indicated above for western blot analysis.

### GST fusion protein

Fusion proteins were purified from transformed *Esherichia coli* (strain BL21) that had been stimulated with isopropyl-β-D-thio-galactopyranoside at a concentration of 0.3 μM. Bacteria were lysed by sonication in solution containing 10 mM Tris-HCl (pH 8.0), 0.5% Nonidet P-40 (NP-40), 100 mM sodium chloride, 20 mM EDTA (pH 8.0) and protease inhibitors. Lysates were then immobilized on glutathione sepharose beads (Pharmacia/GE LifeSciences, Marlborough, MA, USA). Lysates of HEK-293 with and without transfection with T7-Etk/Bmx, colon carcinoma CL1, HCT-116 and HU cells were loaded on these columns. The bound proteins were then washed and sample buffer was added and loaded on SDS–PAGE, followed by immunoblotting with the indicated antibodies and ECL detection.

### GST-*h*PAR_2_-C-tail cloning

The C-tail fragment of *h*PAR_2_, containing 52 amino acids from residue 346 (phenylalanine) to residue 397 (tyrosine), was prepared using PCR amplification (using primers containing *Eco*RI and *Bam*HI restriction sites (respectively, indicated by underlined letters): 5′-CGGAATTCTTTGTTTCACATGATTTCA-3′ and 5′-CGGGATCCATAGGAGGTCTTAACAGT-3′). The resulting DNA fragment was further cut with the appropriate restriction enzymes (*Bam*HI and *Eco*RI) and ligated into the pGEX-2T vector. GST-*h*PAR_2_K378Z-C-tail and GST-*h*PAR_2_K356Z-C-tail were also prepared using the same technique. The suitability of the constructs was confirmed by dideoxy sequencing followed by SDS–PAGE separation, which indicated that the fusion proteins of the C-tails were adequately prepared. The molecular weights of GST fusion proteins were as follows: 27 kD for GST itself, 32.9 kD for the GST-*h*PAR_2_-C-tail, 30.7 kD for the GST-*h*PAR_2_K378Z-C-tail, 28.2 kD for the GST-*h*PAR_2_K356Z-C-tail, 45 kD for GST-PH-Akt and 80 kD for GST-PH-Vav_3_.

### GST-PH-Etk/Bmx

The PH domain in Etk/Bmx was bound to the GST column as previously described^27^. Briefly, GST fusion proteins and His-T7-tagged proteins were expressed in bacteria and purified by using glutathione sepharose (Pharmacia) or Ni^2+^ column (Novagen) as recommended by the manufacturers. The purified GST fusion proteins remained on the glutathione sepharose beads and were then mixed with purified His-T7-tagged PH domain in PBS containing 0.5 mg ml^−1^ bovine serum albumin. After overnight incubation, the beads were collected and extensively washed with cold PBS. The bound proteins were analysed using SDS–PAGE followed by western blot analysis with anti-T7 antibody. The GST-PH-Akt construct was kindly provided by Dr Brian A. Hemmings (Friedrich Miescher Institute). The GST-PH-Vav_3_ construct was kindly provided by Dr Shan Lu (University of Cincinnati College of Medicine).

### HA-tag *wt hPar1* and HA-mutant *hPar1-*7A C-tail constructs

Mutants were designed for insertion of A at the carboxy terminus of PAR_1_ residues 378–384: SSE**CQRYVYS**ILCCKESS to SSE**AAAAAAA**ILCC (named *hPar1*-7A mutant). For HA-tag *wt hPar1* construct PCR primers were designed and added downstream to the ATG start codon. Primers for the HA-tag were as follows: sense: 5′-TACCCATACGATGTTCCAGATTACGCT-3′ and antisense: 5′-AGCGTAATCTGGAACATCTATGGGTA-3′. Replacement of seven residues with Ala (A) at positions 378–384 was performed by synthesis of oligos containing the mutation. Primer sequences were as follows: *hPar1* 7A mutant: sense: 5′-TCTGAGGCTGCTGCTGCTGCTGCAGCTATCTTA-3′ and antisense: 5′-TAAGATAGCTGCAGCAGCAGCAGCAGCCTCAGA-3′. PCR products were then used as primers on an *hPar1* cDNA template to create an extended product of mutations introduced into the full-length sequence. The amplified DNA fragment was digested with *Pin*AI and *Xba*I from PAR_1_ cDNA and was cloned into pcDNA3-*hPar1* plasmid followed by DNA sequencing.

### Placenta tissue collection and preparation

Placental tissues were prepared as previously described^35^. Briefly, the placental tissues were extracted from discarded material provided by patients who voluntarily and legally chose to terminate pregnancy during the first trimester (between 5- and 12-week gestation). Gestational age was determined by the date of the last menstrual period and ultrasound measurement of fetal crown-rump length. All specimens were obtained strictly in adherence with the Hadassah-Hebrew University Institutional Ethics Committee Guidelines (we received informed consent from women who voluntarily and legally chose to terminate pregnancy; Helsinky 0436-13-HMO; serial number 123130).

### First-trimester villous explants in cultures

The preparation and cultivation of villous explants of various first-trimester placentae was performed as described elsewhere^20^,^35^,^36^,^37^. Briefly, placental tissue originating from samples harvested between 10- and 12-week gestation was immediately rinsed in sterile cold PBS and processed within 2 h of collection. Following dissection under the microscope, ∼2–5 mg weight specimens were carefully laid on 200 μl of solid reconstituted undiluted Matrigel substrate (Becton Dickinson, Bedford, MA, USA) in 0.4-μm pore-culture Millicell-CM culture dish inserts (pore size 0.4 μm, Millipore Corp, Bedford, MA, USA). Explants were cultured in DMEM/F-12 supplemented with 100 U ml^−1^ penicillin, 100 μg ml^−1^ streptomycin and 0.25 μg ml^−1^ ascorbic acid, pH 7.4. Villous explants were maintained in culture for 3 days. Viability of the explants was assessed by adherence to Matrigel and emerging EVTs breaking from the tips as observed under a phase microscope. After 24 h in culture, explants were treated with thrombin (1 U ml^−1^), TFLLRN (100 μM) and SLIGKV (100 μM), and were transfected or not with *wt hPar1, hPar1 7A* mutant or with *wt hPar2* and mutant *hPar2* H349A viral vectors. After 72 h in culture, the Matrigel cast cylinders with the EVTs were detached from the base of the insert cells by fine dissection under the microscope, fixed in 4% paraformaldehyde, embedded in paraffin and further processed for histochemical tissue analysis. Each experiment was carried out in triplicate using samples originating from eight different placental sets.

### EVT invasion assessment

We assessed the H&E-stained serial sections of the EVT. We limited our study to the trophoblasts that penetrated through the Matrigel but remained on the surface of the polycarbonate membrane, that is, within the cast^35^,^36^,^37^. These were visualized on sequential slices of the entire Matrigel cast by haematoxylin staining. Slides were photographed using the digital image capture software and all specimens were evaluated by two authors (S.G.-G. and M.M.). Depth of invasion was assessed for each experiment (disappearance of trophoblast cells on the serial sections). For each set of experimental conditions, the total number of EVT cells was counted and the mean±s.e.m./high power field (HPF) was calculated for five randomly selected microscope fields (magnification × 40) at the deepest common level of invasion.

### Statistical analysis

At the deepest common level of invasion, the total EVT cells at five randomly selected microscope fields were counted, for each experimental condition and the mean±s.e.m./HPF calculated. The data were statistically evaluated using analysis of variance Tukey honest significant difference (HSD) test of multiple comparison showing a *P* value of 0.0001 within groups. The mean difference is significant at the 0.05 level.

## Additional information

**How to cite this article:** Kancharla, A. *et al.* PH motifs in PAR_1&2_ endow breast cancer growth. *Nat. Commun.* 6:8853 doi: 10.1038/ncomms9853 (2015).

## Supplementary Material

Supplementary Information Supplementary Figures 1-12 and Supplementary Tables 1-2
