# Supplementary material for: PH motifs in PAR1&2 endow breast cancer growth
Source: Nat Commun. 2015 Nov 24;6:8853. doi: 10.1038/ncomms9853 (PMC4673491; doi:10.1038/ncomms9853)
Supplement: Supplementary Information — Supplementary Figures 1-12 and Supplementary Tables 1-2 [file ncomms9853-s1.pdf]

Supplementary Information

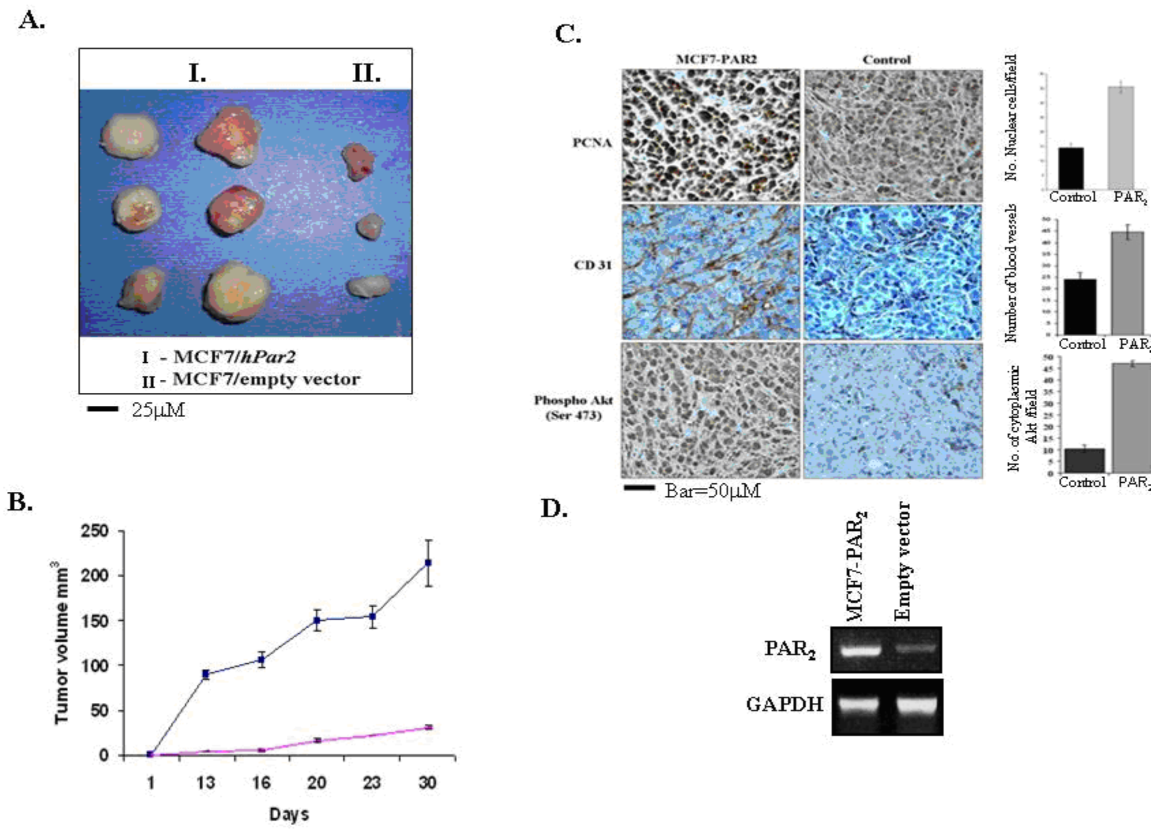

Supplementary figure 1: **PAR_2 elicits mammary gland tumors in a xenograft mouse model. A**. Morphological appearance of the tumors. I. Large and vascularized tumors obtained by orthotopic injection of MCF-7 cells stably overexpressing *hPar2* (MCF7/*hPar2*) after the inoculation of estrogen pellets, and II. mock transfected cell injected (MCF7/empty vector). **B**. Tumor volume of the two groups is shown. **C.** Immunohistological staining of the tumor sections. PCNA antibody was used to evaluate the extent of cell proliferation in tumor tissue sections. Strong nuclear staining was observed in *wt* PAR_2 (large tumors) as compared with the mock transfected PAR_2 clone (small tumors), which showed a low level of staining. Similarly, when anti-CD31 antibody was applied to the tissue sections, a high level of CD31 staining was seen in the PAR_2 tumors indicating a high level of vascularity, as compared with low levels in the control tissue section. PAR_2-driven tumors exhibited elevated cytoplasmic levels of phosphotylated Akt (Ser473) as compared with low levels in their control counterparts. **D**. RT-PCR analyses of the injected clones. Levels of *hPar2* in the stable clones as compared with a housekeeping gene GAPDH are shown.

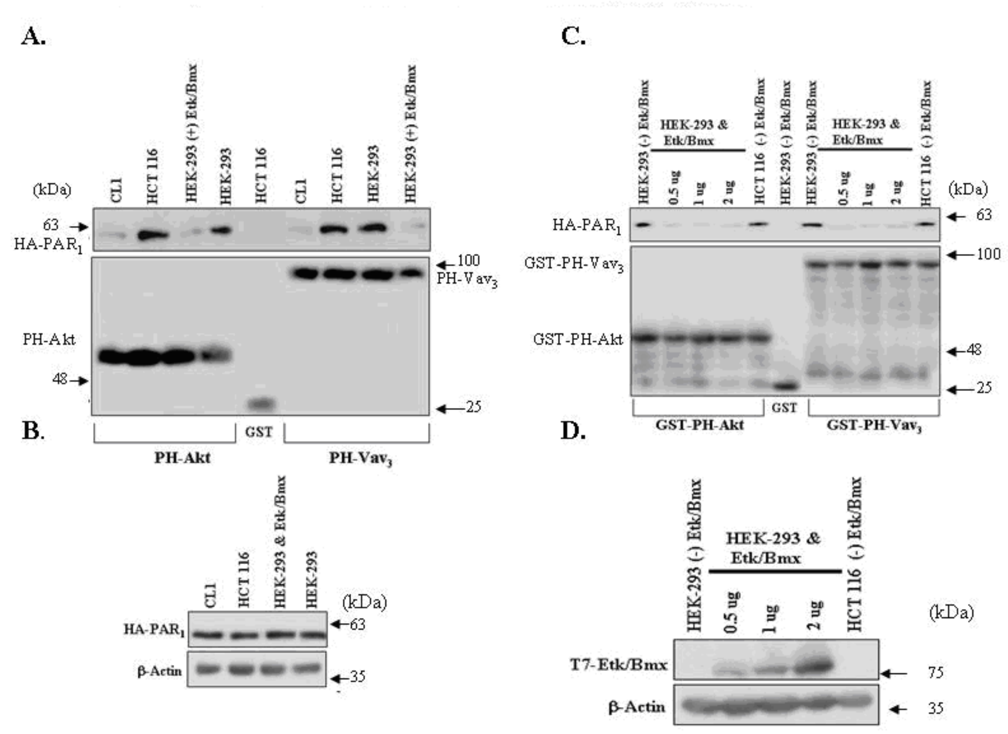

Supplementary figure 2: Akt and Vav3-PH domain specifically associate with PAR_1. A. **GST-PH-Akt or GST-PH-Vav3 pull down assay for PAR_1.** Tumor cell lines: CL1, HCT116, and HEK-293T cells were transfected with HA-*hPar1*. HEK 293T cells were transfected either with or without Etk/Bmx. Cell lysates were applied on GST-PH-Akt and GST-PH-Vav3 columns, and Western blot analysis was performed using anti-HA antibodies. GST-PH-Akt and GST-Vav3 were shown to bind to HA-tag-PAR_1 in cells that did not express Etk/Bmx (e.g., HCT-116 and HEK 293T cells). In contrast, HEK293T cells transfected to express Etk/Bmx did not bind to either Akt or Vav3. B. The level of HA-PAR_1 following transfection is shown with β-actin levels used as loading controls. C. HEK293T cells were transfected with increasing concentrations of Etk/Bmx and HA-*hPar1*. Cell lysates were applied on GST-PH-Akt and GST-PH-Vav3 columns. No binding was observed when Etk/Bmx was expressed in these cells. Binding to either Akt or Vav3 was abrogated also at the lowest concentration of Etk/Bmx used (e.g., 0.5 μg). D. Levels of T7-Etk/Bmx following transfection are shown. β-actin was used as loading control.

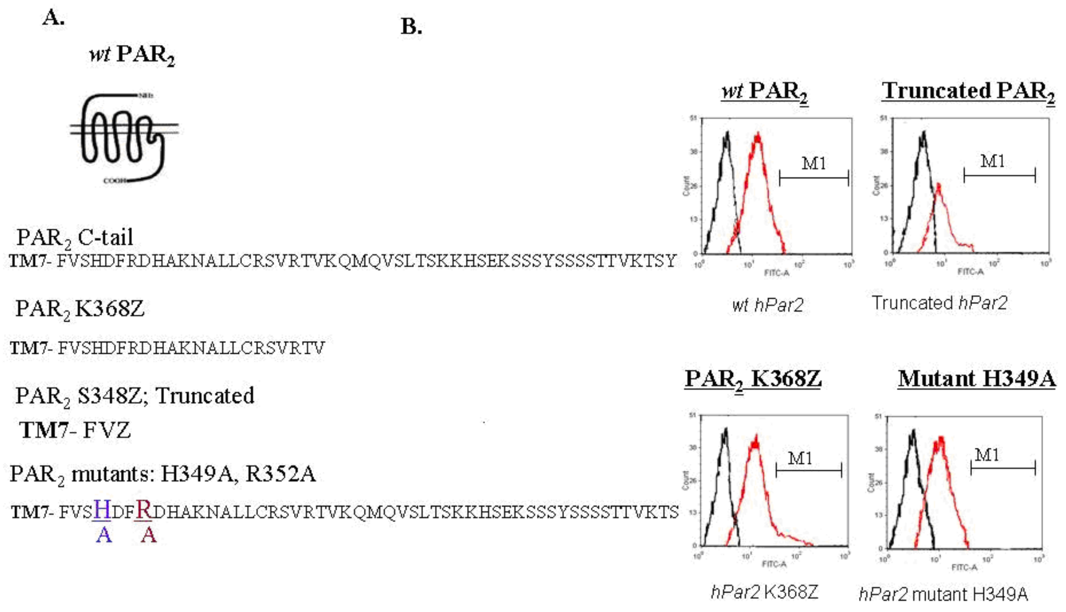

Supplementary figure 3: Surface expression of *hPar2* constructs. A. Schematic presentation of *wt* PAR_2 and sequence alignment of the various C-tail constructs. **B.** FACS *(fluorescence-activated cell sorting)* analysis. Findings from flow cytometry analysis of surface-expressed *wt hPar2*, truncated *hPar2* (S348Z), K368Z and mutant H349A are shown. Deletion constructs K390Z, K378Z (data not shown) were also evaluated. Constructs were transiently expressed in HU cells and surface expression was determined by flow cytometry analysis using anti-PAR_2 ab (SAM11 1–2 μg/ml; Santa Cruz Biotechnology, Santa Cruz, CA, USA) that were directed to detect cell surface levels of PAR_2. Analyses were performed on intact cells. Construct expression levels were similar (somewhat slightly lower level of truncated PAR_2) regardless of whether *wt* or other deletion constructs or mutants were analyzed. The first balck peak represents the isotype control antibody alone; the red peak represents the PAR_2 antibody.

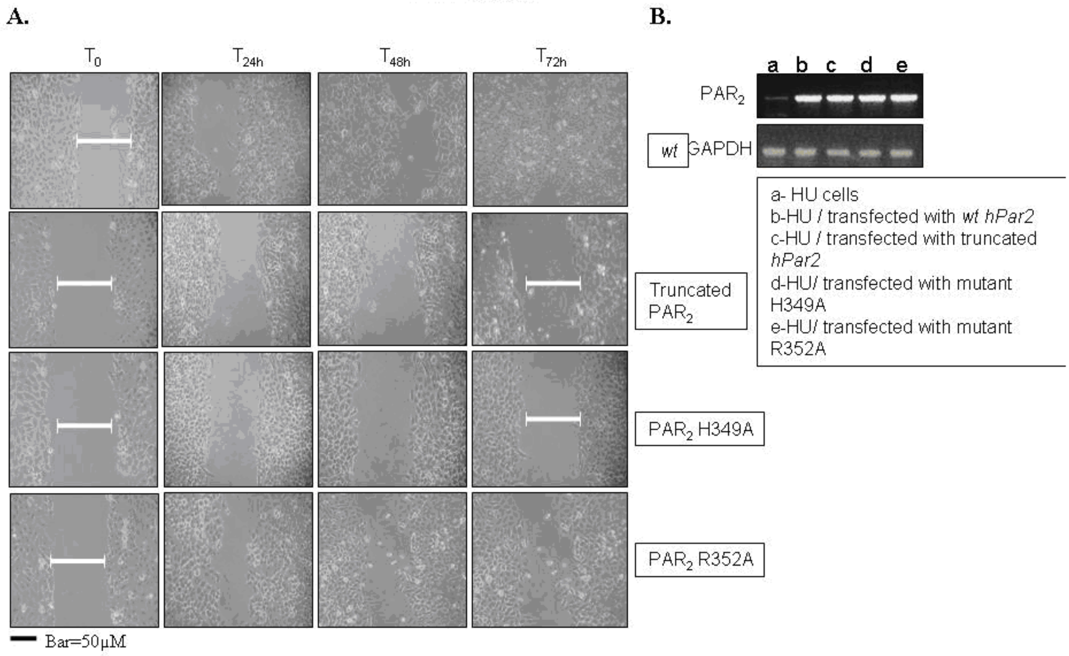

Supplementary figure 4: Cell-wound migration assay. HU cells were ectopically transfected to express either *wt* or deleted and mutated *hPar2* constructs. The following constructs were used: *wt hPar2,* truncated *hPar2,* PAR_2 mutant H349A, and mutant PAR_2 R352. Following transfection the cells were grown to confluence then serum-deprived for 24 hr. After serum deprivation, a defined wound was applied to the culture dishes. Extensive rinsing of the plate dishes were then applied to remove residual debris and the cells were refed with medium containing low serum and PAR_2 activating peptide SLIGKV for the indicated time periods. **A.** Phase contrast images were taken 24h, 48h, and 72h later to assess cell migration into the wounds. Images are representative of two independent experiments performed in triplicate. Note that wound is nearly completed by 72h in the *wt* triggered PAR_2 culture, while, in contrast, the wound remains wide open in the presence of either truncated PAR_2 or the PAR_2 mutant H349A. In the presence of PAR_2 mutant R352A, the cells are migrating toward the middle of the wound, albeit to a slower pace than *wt* PAR_2. Data shown are representative of three independent experiments. **B.** Levels of various PAR_2 forms in HU cells before and after different transfection treatment. RT-PCR analyses.

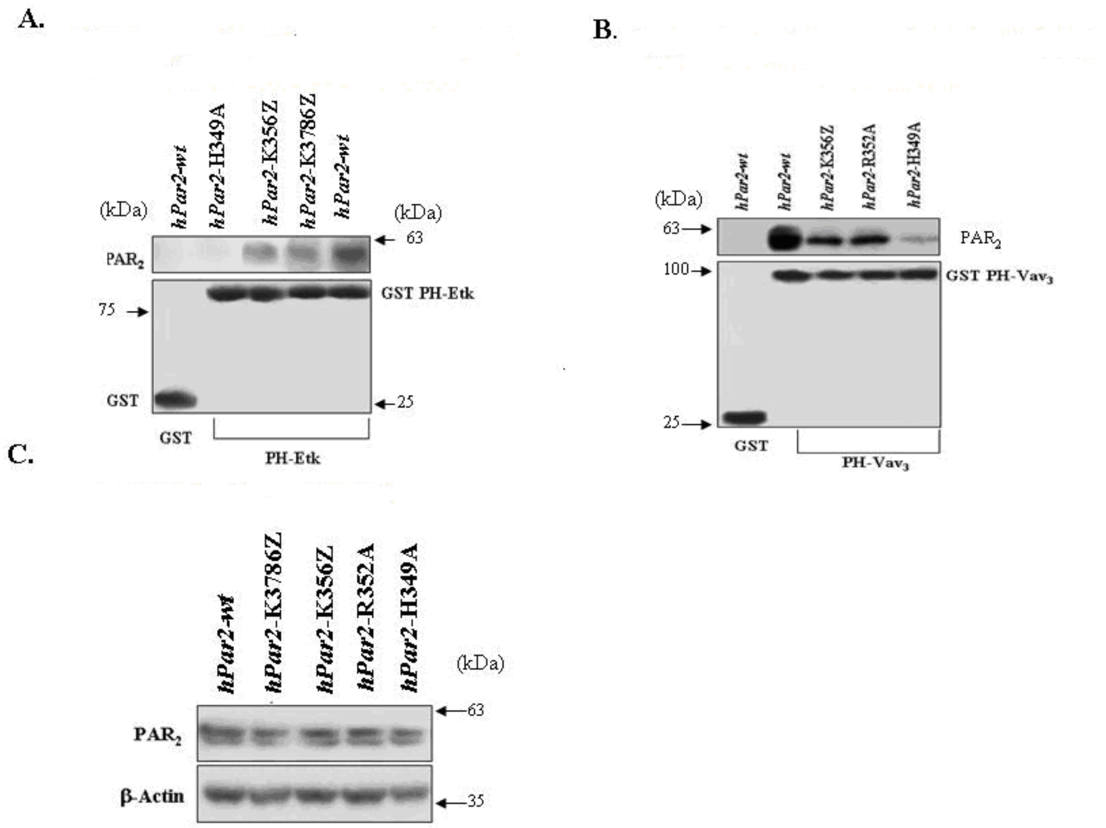

Supplementary figure 5: Elucidation of the PAR_2 minimal PH-domain region. A. **Binding to PH-Etk/Bmx**. Nearly normal HU cells (do not express PAR_2) were transiently transfected, either to overexpress *wt hPar2*, or the deleted *hPar2* C-tails K368Z and K356Z, as well as mutant H349A. Cell lysates were applied on a GST-PH-Etk/Bmx pull-down assay. While specific binding was observed upon application of either *wt* or deleted C-tail constructs, the mutant H349A did not show any binding similar to that seen with *wt hPar2* application on GST beads alone. **B. Binding to PH-Vav3**. A similar pattern of binding was observed when cell lysates were applied on GST-PH-Vav3 columns. Binding was seen in the *wt* and deletion constructs of PAR_2 C-tails but not with the mutant H349A. **C.** Levels of transfection. Western blot analysis showing the transfection efficiency of the various constructs.

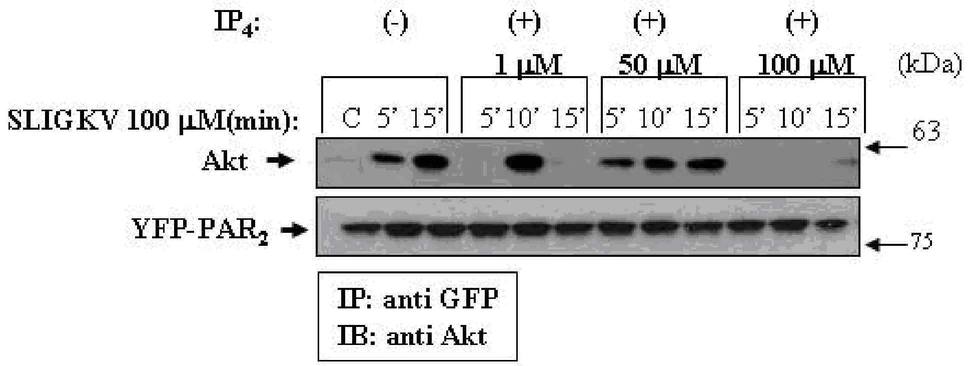

Supplementary figure 6: IP_4 inhibits PAR_2 association with PH-Akt. HU cells were transiently transfected with YFP-*hPar2* and incubated, or not, with increasing concentrations of IP_4 (Echelon Biosciences Inc. UT, USA) 15 minutes prior to SLIGKV PAR_2 activation. Immunoprecipitation was carried out using anti-GFP ab and levels of Akt bound to PAR_2 were detected following application of anti Akt (Cell Signaling Technology Inc. MA, USA) to the immunopreciptants. Levels of PAR_2 were detected by application of anti-GFP antibodies (Cell signaling Technology Inc. MA, USA).

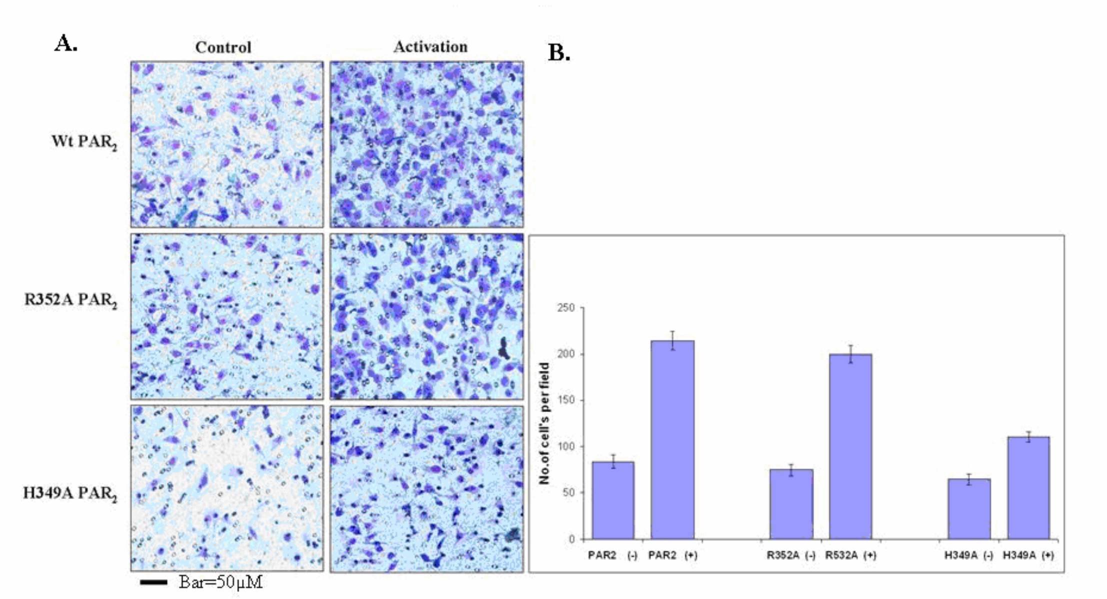

Supplementary figure 7: PAR_2 mutant H349A fails to induce Matrigel invasion. **A.** Matrigel invasion assay was performed following SLIKGV PAR_2 activation of HU clones overexpressing either *wt hPar2*, or PAR_2 mutants R352A or H349A. Induced Matrigel invasion levels were seen in the *wt* PAR_2-activated clone and mutant R352A. In contrast, SLIKGV activation of PAR_2 H349A mutant failed to induce Matrigel invasion, suggesting inability of PH-domain binding association. B. Histograms representing the extent of Matrigel invasion are shown (unpaired Student's *t*-test). Representative of three independent experiments performed in triplicate. Error bars, $\pm$ SD, $^{**}p<0.009$.

Supplementary Fig. 8:

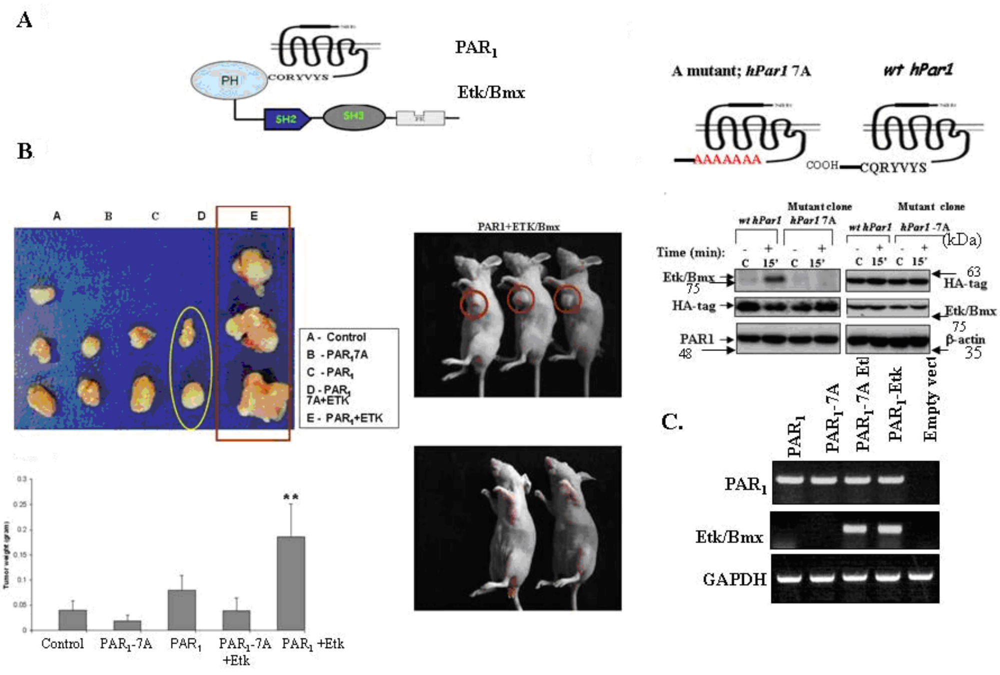

Supplementary figure 8: PAR_1- mammary tumors are inhibited in PAR_1 7A mutant. A. Schematic presentation of PAR_1-Etk/Bmx**.** Schematic presentation of *wt* and mutant *hPar1* 7A, of alanine replacement, A in the putative PH-domain C-tail binding site. Left panel. Co-association between *wt* PAR_1 and Etk/Bmx in MCF-7 clones overexpressing *hPar1* and Etk/Bmx. In contrast, no association is seen when the mutant *hPar1* 7A and Etk /Bmx *clone* are used. Right panel. Transfection efficiency of the clones used. **B.** Large and vascularized tumors are obtained following orthotopic injection of MCF-7 clones stably expressing *wt hPar1* and *etk/bmx*. In contrast, no or only very small tumors are seen following injection of cells stably expressing the PAR_1 7A mutant, which is incapable of associating with Etk/Bmx or *etk/bmx*. These findings are similar to results following injection of control mock-transfected MCF-7 cells. Mice carrying *hPar1/etk/bmx* large tumors or *hPar17A/etk/bmx* small or no tumors. Histograms show statistically significant outcome. Error bars show +/- SD of mean and the *P* value was determined (*<0.006 *P< 0.003; Chi-square test).A representative image of the mice is shown. **C.** RT-PCR analysis showing transfection levels of the various MCF-7clones.

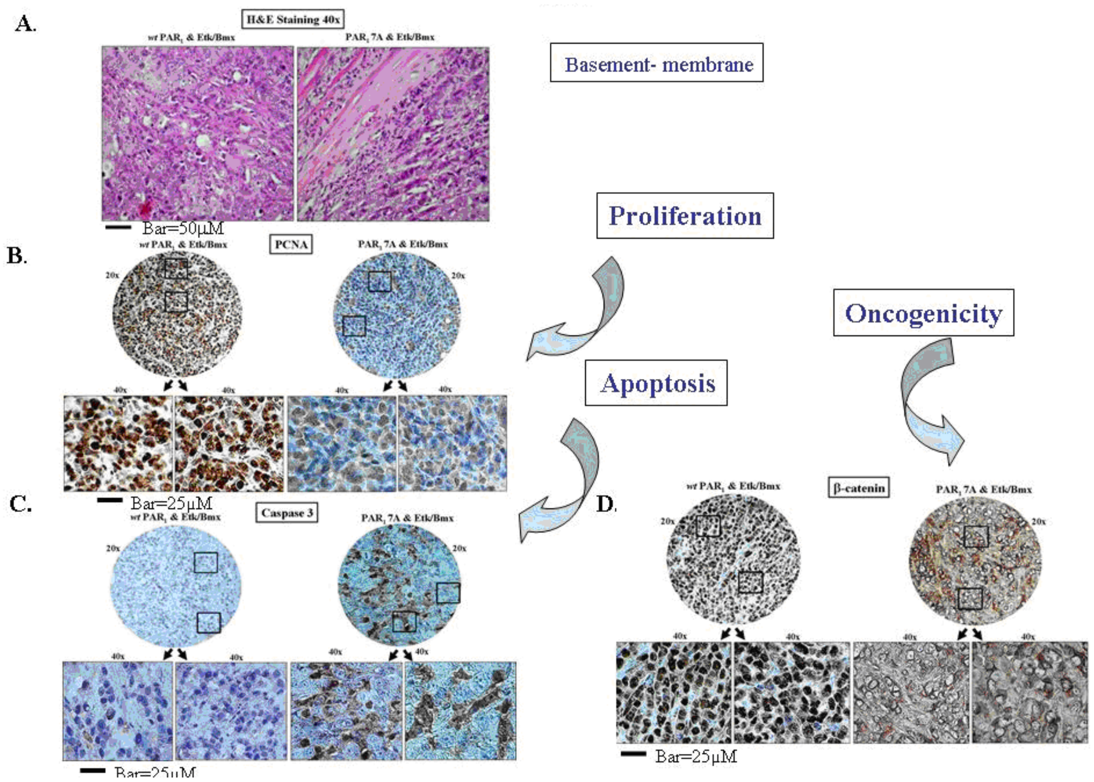

Supplementary figure 9: Immunohistology of PAR_1/Etk/Bmx- and PAR_1-7A/Etk/Bmx-tumors. A. Hematoxylin and eosin (H&E) staining of large PAR_1/Etk tumors and small PAR_1-7A/Etk tumor sections. B. PCNA antibody was used to evaluate cell proliferation levels in the tissue sections. Strong nuclear staining was observed in *wt* PAR_1 & Etk/Bmx sections (large tumors) compared to PAR_1-7A-Etk/Bmx (small tumors), which showed low-level or no staining. **C**. Caspase 3 antibody was used to detect levels of apoptosis. A large number of apoptotic cells were obseved in sections obtained from PAR_1-7A and Etk/Bmx small tumors, as evidenced by high levels of caspase 3 in the cell cytoplasm. No staining was observed in *wt* PAR_1-Etk/Bmx tumors, indicating the absence of apoptotic cells in the tumor sections. **D**. β-catenin antibody was used to evaluate oncogenesis. Strong nuclear staining was observed in *wt* PAR_1-Etk/Bmx tumors, whereas PAR_1-7A-Etk/Bmx exhibited weak cytoplasmic β-catenin staining.

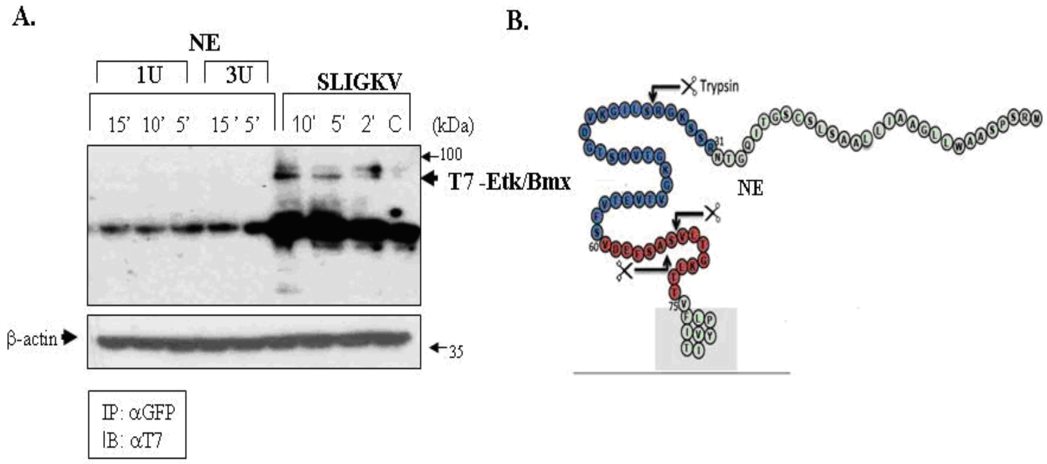

Supplementary figure 10: NE does not induce association of PH-Etk/Bmx with PAR_2. A. Naïve HU cells were transiently transfected with YFP-*hPar2* plasmid and T7-tagged *etk/bmx*. Immunoprecipitation was performed following PAR_2 activation using anti-GFP antibodies (to detect PAR_2) and immunoblotting with anti-T7 for Etk/Bmx detection (T7 tagged Etk/Bmx). Effective co-immunoprecipitation was seen following SLIGKV PAR_2 activation of *YFP- hPar2* but not following NE activation. As control, equal levels of β-actin are shown. **B.** Scheme showing the PAR_2 N-terminus with arrows marking the major cleavage sites of trypsin and NE.

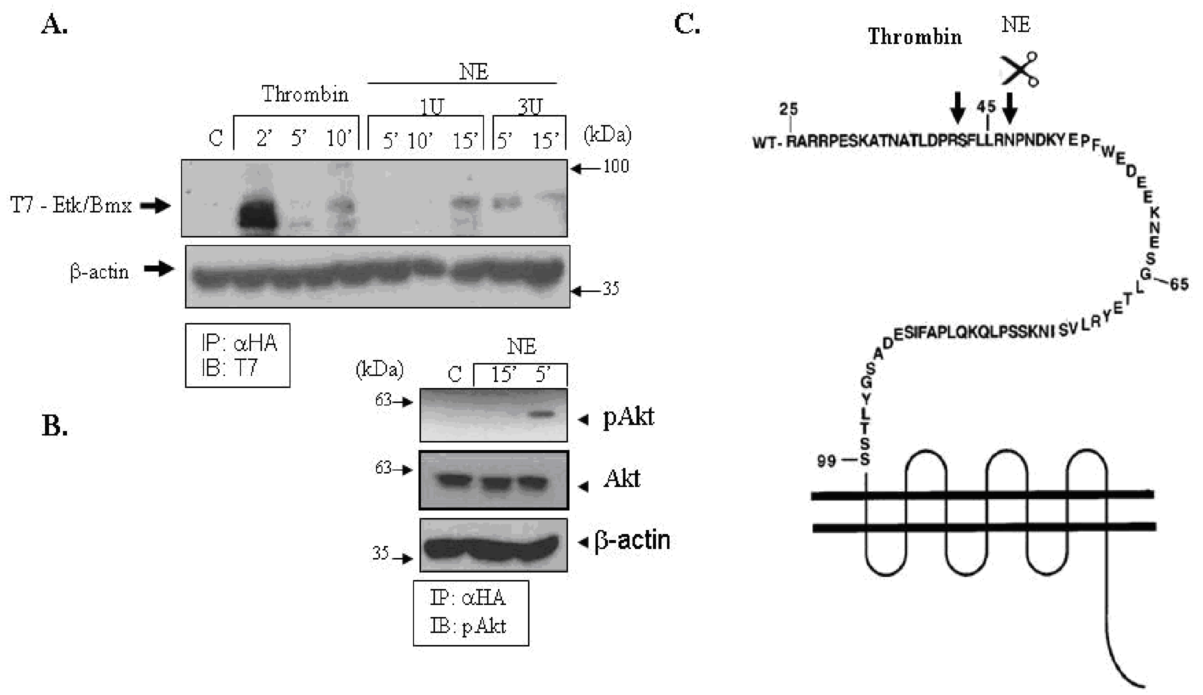

Supplementary figure 11: NE induces association of PH-Etk/Bmx and PH-Akt with PAR_1. A. Naïve HU cells were transiently transfected with HA-*hPar1* plasmid and T7-tagged *etk/bmx*. Immunoprecipitation was performed following PAR_1 activation using anti-HA antibodies (to detect PAR_1) and immunoblotting with anti-T7 for Etk/Bmx detection (T7 tagged Etk/Bmx). Effective co-immunoprecipitation was seen following thrombin/TFLLRN PAR_1 activation of HA-*hPar1*, and also following NE activation, although to a somewhat diminished extent. As control equal levels of β-actin are shown. **B.** Scheme showing the PAR_1 N-terminus with arrows showing the major cleavage sites of Thrombin and NE.

Supplementary Fig. 12:

A.

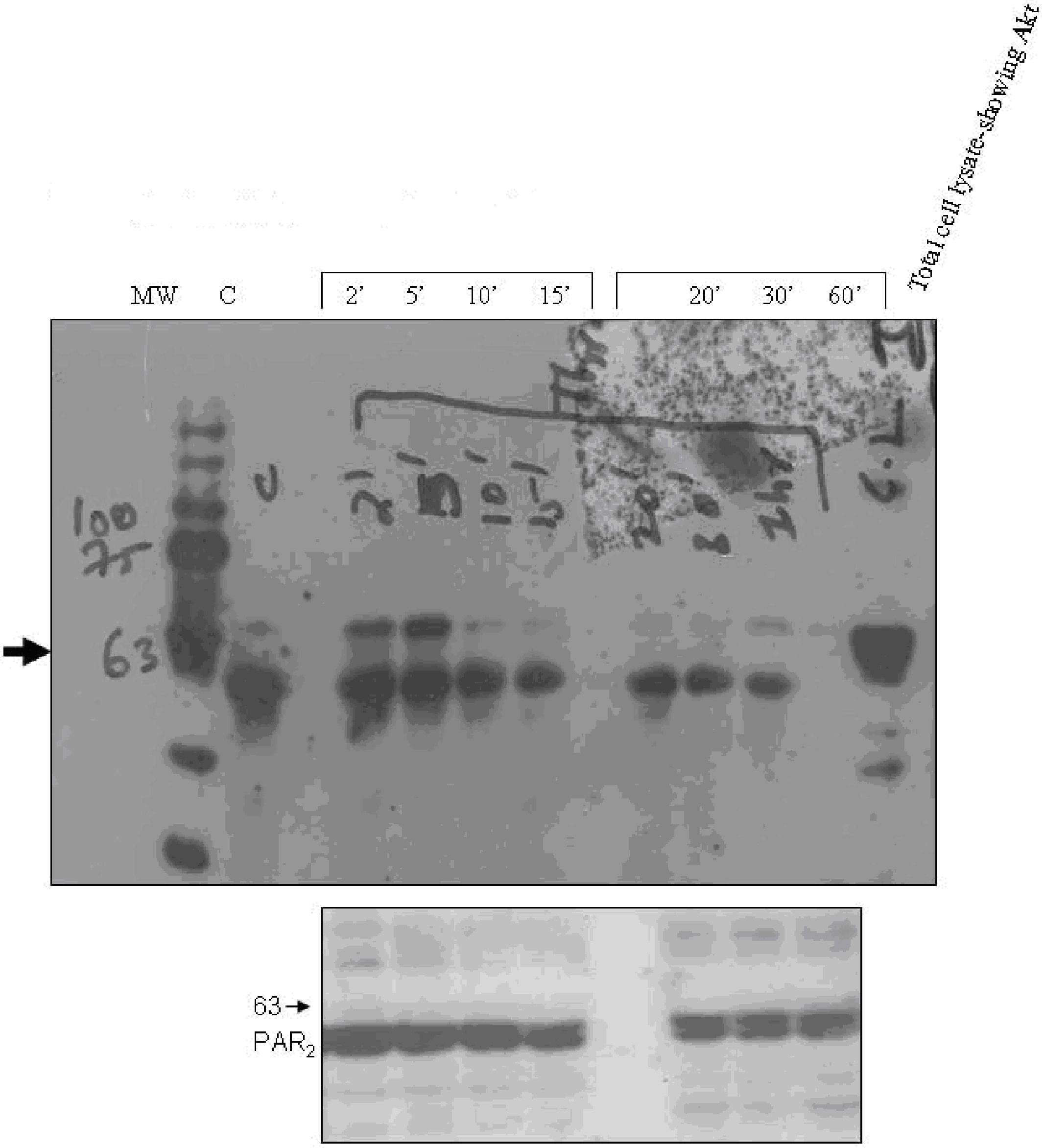

Supplementary Fig. 12: Uncropped western blots related to Figs 1B**,** D, G; 2B**,** E, G; 3B, 4A, D and Supplementary Figs. 2, 6, 10 and 11.

B.

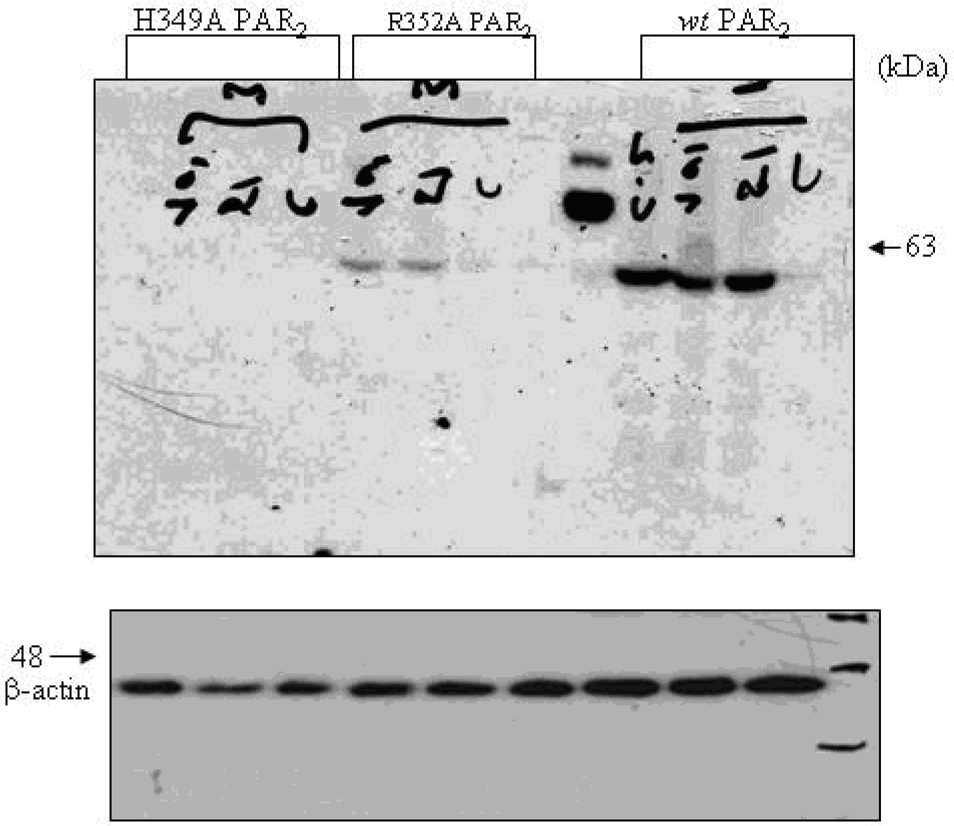

Supplementary Fig. 12 continued

C.

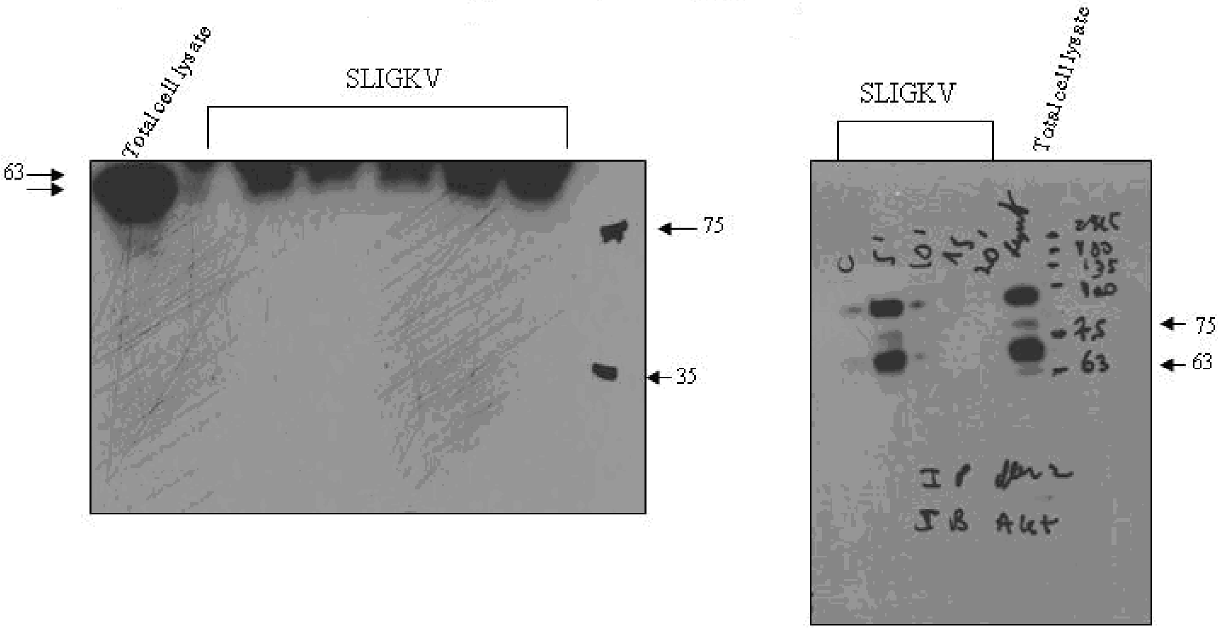

Supplementary Fig. 12 continued

D.

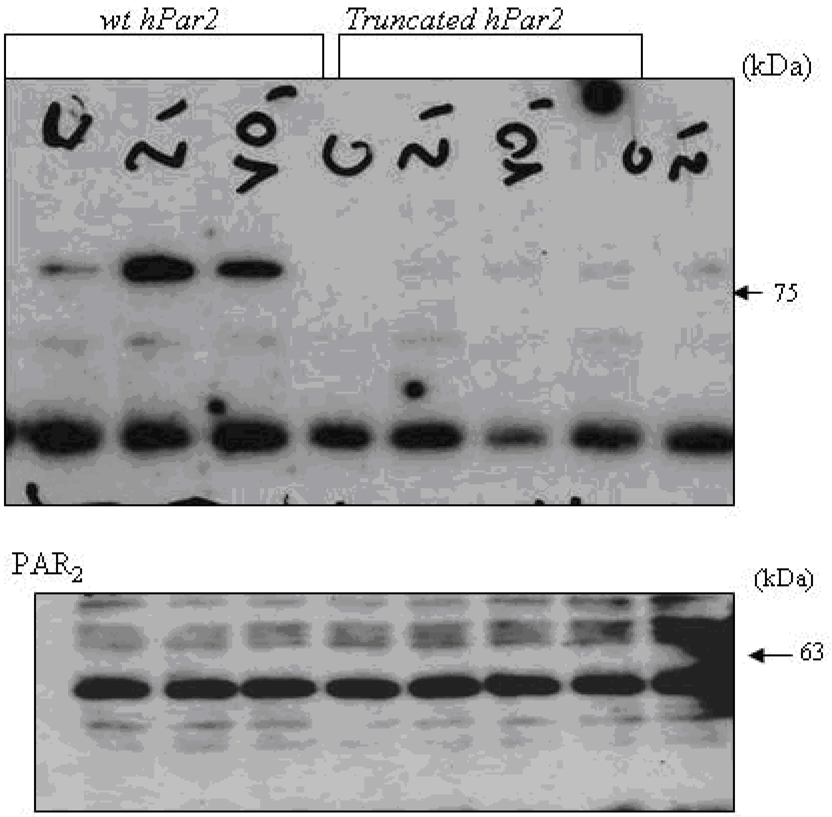

Supplementary Fig. 12 continued

E.

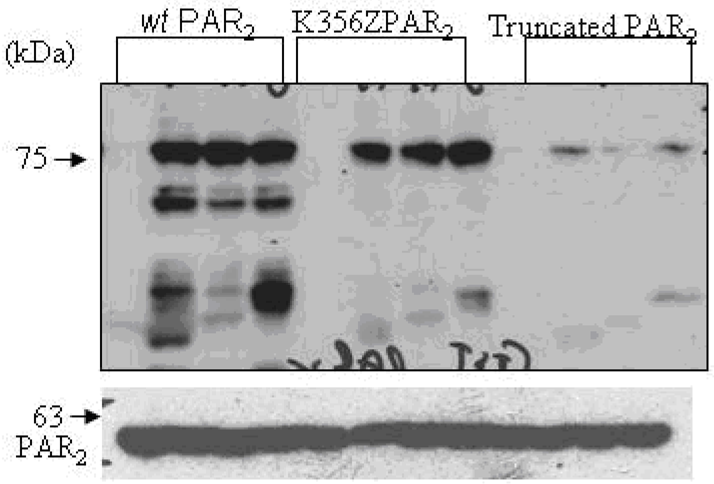

Supplementary Fig. 12 continued

F.

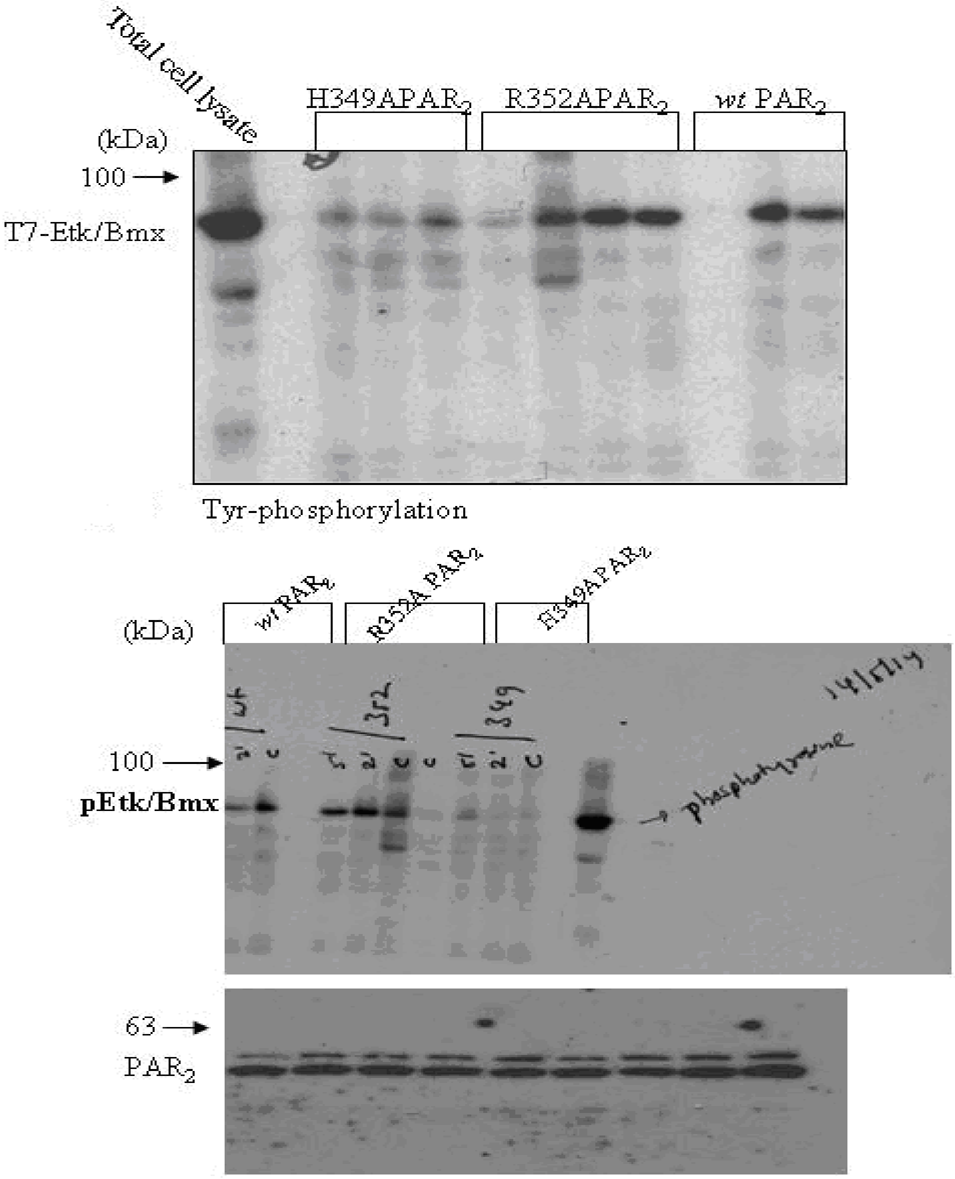

Supplementary Fig. 12 continued

G.

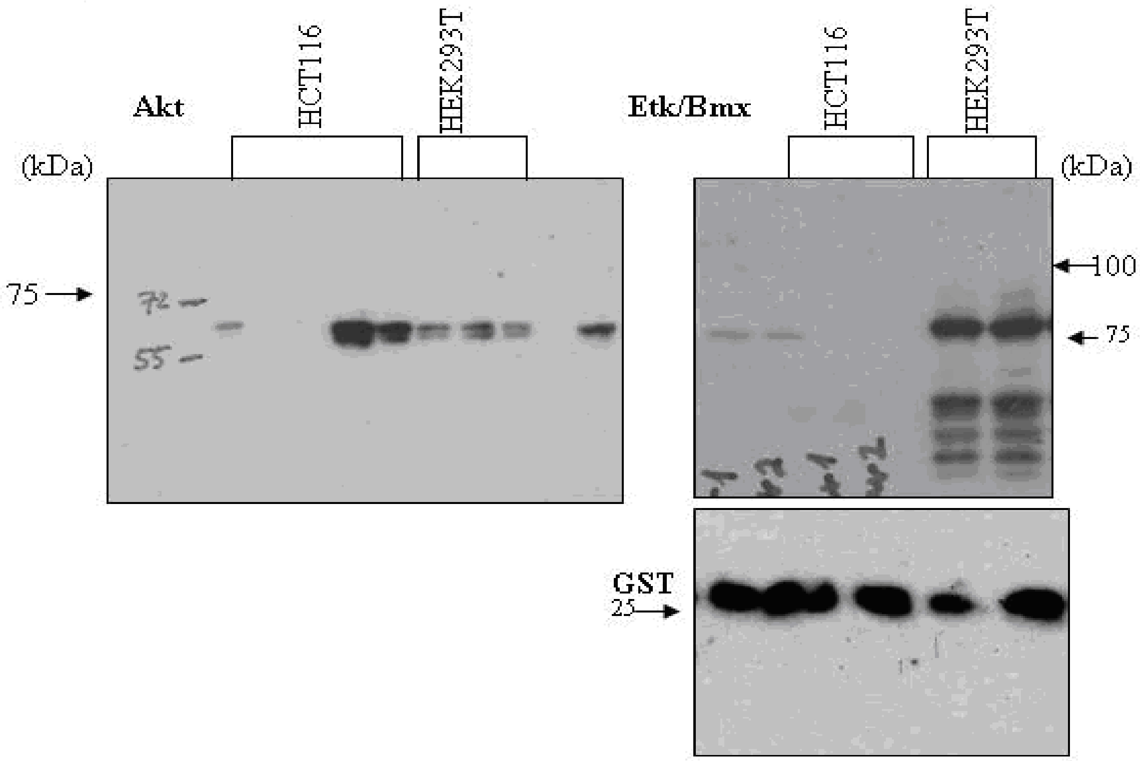

Supplementary Fig. 12 continued

H.

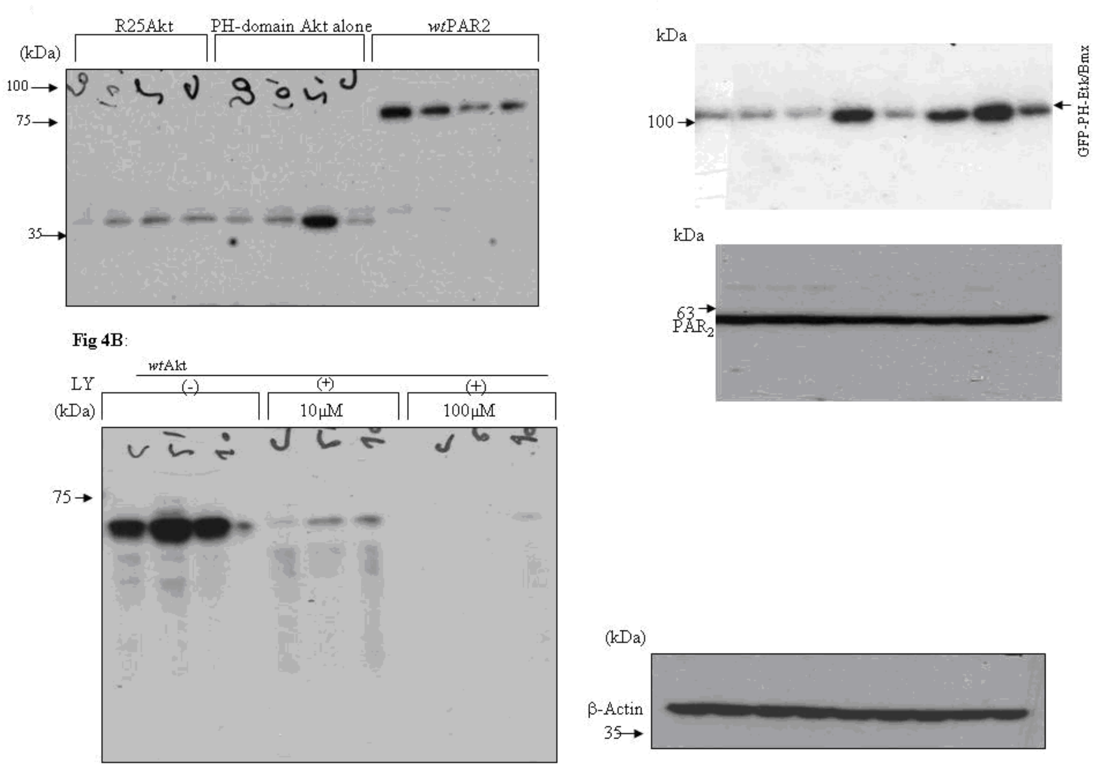

Supplementary Fig. 12 continued

I.

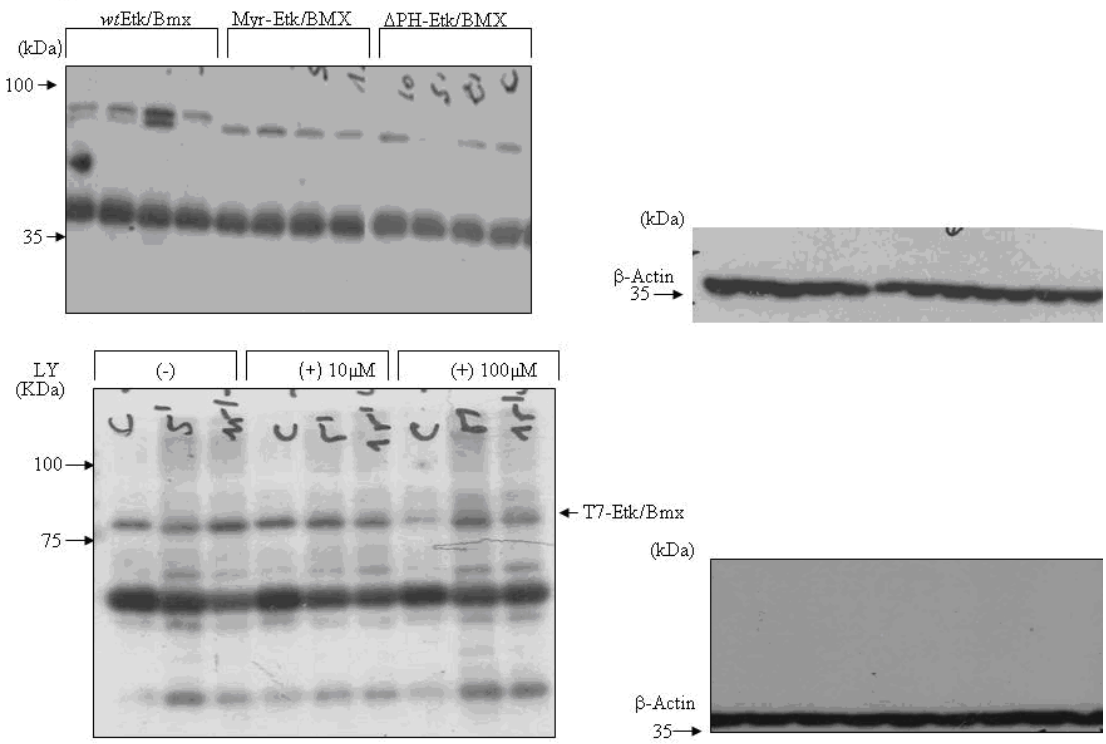

Supplementary Fig. 12 continued

SFig. 12J:

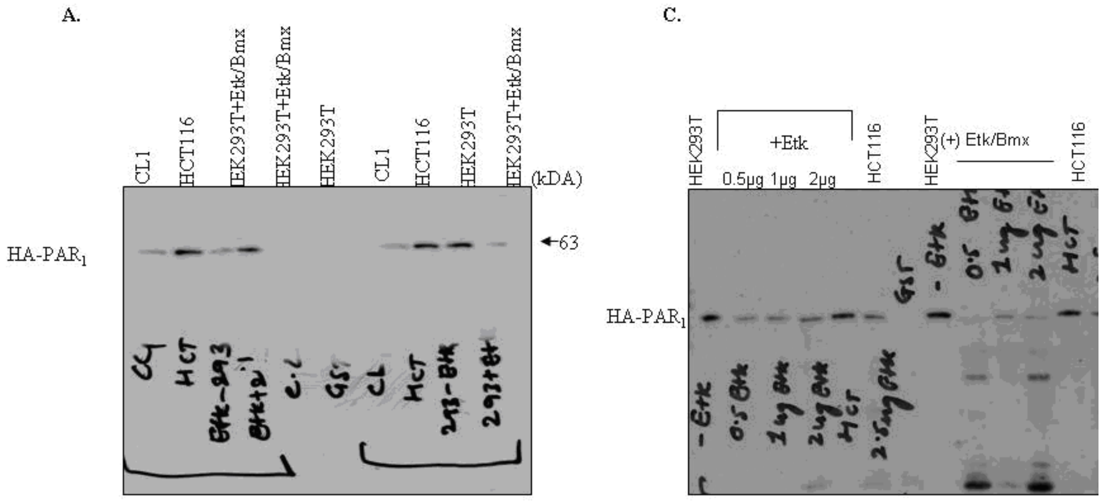

Supplementary Fig. 12 continued

SFig. 12K:

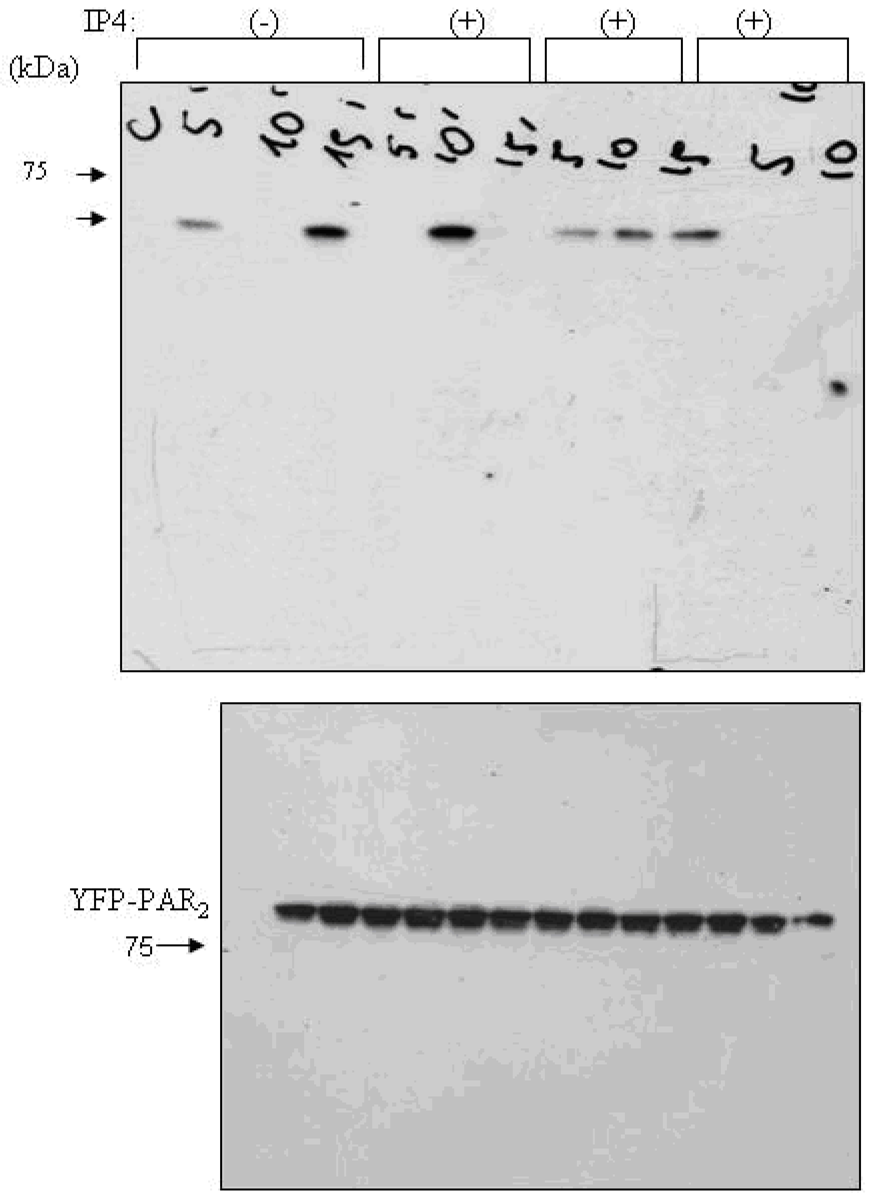

Supplementary Fig. 12 continued

SFig. 12L:

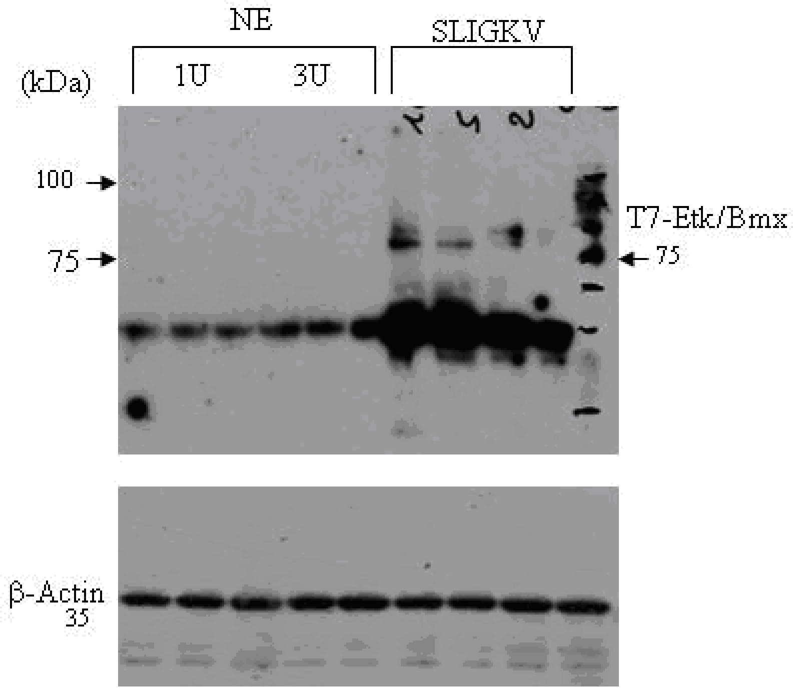

Supplementary Fig. 12 continued

SFig. 12M:

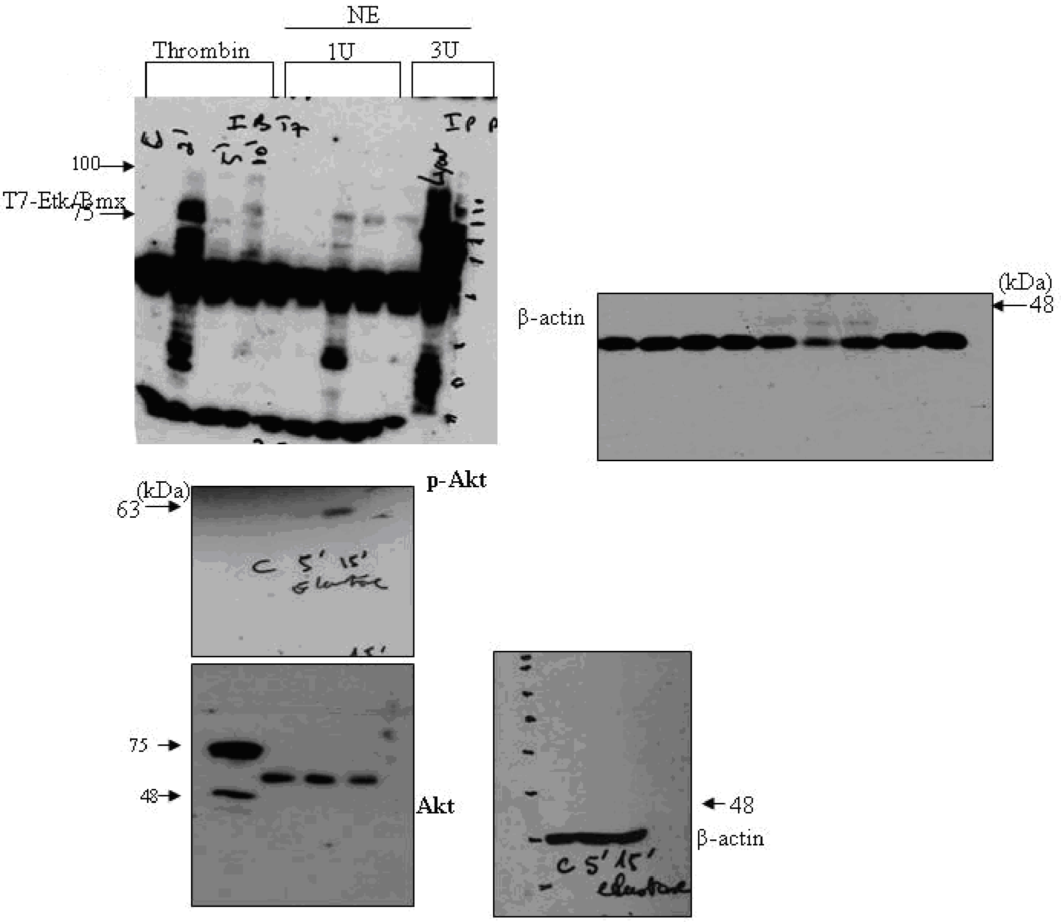
NE
Thrombin
1U
3U
100
T7-Etk/Bmx
75
β-actin
(kDa)
48
(kDa)
63
p-Akt
75
48
Akt
48
β-actin

Supplementary Table 1:

PAR2 C-tail deleted constructs and mutants

**PAR_2 C-tail *wt***	TM7- FVHDFRDHAKNALLCRSVRTVKQMQVSLTSKKHSRKSSSYSSSSTTV
**PAR_2K390Z**	TM7-FVHDFRDHAKNALLCRSVRTVKQMQVSLTSKKHSRKSSSYSSSS**Z**
**PAR_2K378Z**	TM7-FVHDFRDHAKNALLCRSVRTVKQMQVSLTSK**Z**
**PAR_2K368Z**	TM7-FVHDFRDHAKNALLCRSVRTV**Z**
**PAR_2K356Z**	TM7-FVHDFRDHA**Z**
**Truncated PAR_2 S348Z**	TM7-FVZ
**R352A**	TM7-FVHDF**A**DHA**Z**
**H349A**	TM7-FV**A**DFRDHA**Z**

Supplementary Table 2 Primer sequences

**Primers used for insertion of stop codons along *hPar2* C-tail**	
S390Z	5’- GCTCTTACTCTTCAAGT**TGA**ACCACTGTTAAGACCTCC- 3’
K378Z	5’CCCTCA CC TC AAAG**TAA**CACTCCAGGAAATCCAGC-3’
K368Z	5’-GCCGAAG TGTCC GCACTGT A**TAG**CAGATGCAAGTATCCC-3’
K356Z	*5’*-GGGATCATGC A**TAG**AACGCTCTCCTTTGCCGAAGTGTCCGC-3’
Truncated hPar2 (S348Z)	CGACCCCTTTGTCTATTACTTTGTT**TCA**CATGATTTCAGGG
**Primers used for generation of mutants R352A and H349A**	
**R352A**	Forward: 5’-CACATGATTTCgcGGcTgcTGCAAAGAACGCTCTCCTTTGCCG-3
	Reverse: 5’CGGCAAAGGAGAAGCGTTCTTTGCAgcAgCCgcGAAATCATGTG 3’
**H349A**	Forward: 5’ CCCCTTTGTCTATTACTTTGTTTCAgcTGcTgcCAGGGATCATGC- 3’
	Reverse: 5’- GCATGATCCCTGGCAGCAGCTGAAACAAAGTAATAGACAAAGGGG-3’
